# Application of machine learning in predicting hospital readmissions: a scoping review of the literature

**DOI:** 10.1186/s12874-021-01284-z

**Published:** 2021-05-06

**Authors:** Yinan Huang, Ashna Talwar, Satabdi Chatterjee, Rajender R. Aparasu

**Affiliations:** Department of Pharmaceutical Health Outcomes and Policy, College of Pharmacy, University of Houston, 4849 Calhoun Road, Health & Sciences Bldg 2, Houston, TX 77204 USA

**Keywords:** Machine learning, Hospital readmission, Scoping review, Prediction

## Abstract

**Background:**

Advances in machine learning (ML) provide great opportunities in the prediction of hospital readmission. This review synthesizes the literature on ML methods and their performance for predicting hospital readmission in the US.

**Methods:**

This review was performed according to the Preferred Reporting Items for Systematic Reviews and Meta-Analysis Extension for Scoping Reviews (PRISMA-ScR) Statement. The extraction of items was also guided by the Critical Appraisal and Data Extraction for Systematic Reviews of Prediction Modelling Studies (CHARMS). Electronic databases PUBMED, MEDLINE, and EMBASE were systematically searched from January 1, 2015, through December 10, 2019. The articles were imported into COVIDENCE online software for title/abstract screening and full-text eligibility. Observational studies using ML techniques for hospital readmissions among US patients were eligible for inclusion. Articles without a full text available in the English language were excluded. A qualitative synthesis included study characteristics, ML algorithms utilized, and model validation, and quantitative analysis assessed model performance. Model performances in terms of Area Under the Curve (AUC) were analyzed using R software. Quality in Prognosis Studies (QUIPS) tool was used to assess the quality of the reviewed studies.

**Results:**

Of 522 citations reviewed, 43 studies met the inclusion criteria. A majority of the studies used electronic health records (24, 56%), followed by population-based data sources (15, 35%) and administrative claims data (4, 9%). The most common algorithms were tree-based methods (23, 53%), neural network (NN) (14, 33%), regularized logistic regression (12, 28%), and support vector machine (SVM) (10, 23%). Most of these studies (37, 85%) were of high quality. A majority of these studies (28, 65%) reported ML algorithms with an AUC above 0.70. There was a range of variability within AUC reported by these studies with a median of 0.68 (IQR: 0.64–0.76; range: 0.50–0.90).

**Conclusions:**

The ML algorithms involving tree-based methods, NN, regularized logistic regression, and SVM are commonly used to predict hospital readmission in the US. Further research is needed to compare the performance of ML algorithms for hospital readmission prediction.

**Supplementary Information:**

The online version contains supplementary material available at 10.1186/s12874-021-01284-z.

## Background

The continuing efforts to reduce hospital readmission rates in the US have largely been driven by the great understanding of readmission rates among individuals and the associated costs to the health system. Hospital readmissions are common for patients discharged following hospitalization in the US, especially among the population with baseline comorbidities [1], and the elderly population group [2]. Readmission causes a significant financial burden for public and private payers [3, 4]. In response to such problems, multiple initiatives have been mandated through the Affordable Care Act in the efforts to reduce hospital readmissions [5]. The Hospital Readmission Reduction Program (HRRP) that penalizes hospitals with higher than average readmission rates is among the most prominent initiatives [6, 7]. In addition, reduction in readmission rates has been recognized as a part of national strategies for quality improvement through other incentives of health care policies [8, 9]. Therefore, models for predicting readmission risk are in great demand, and these tools could help to identify and reduce readmission with a goal to improve overall patient care and reduce healthcare costs.

The Centers for Medicare and Medicaid Services (CMS) uses risk-standardized readmission models based on hierarchical logistic regression [10–13]. Meanwhile, there has been growing interest among payers in developing models for readmission risk to reduce costs and improve care, given readmission reduction is  a part of quality of care imperatives. Machine Learning (ML) techniques are gaining popularity for clinical utility amid the growing availability of healthcare data [14–16]. ML is a powerful method of data analysis that is based on the concepts of learning and discovering data patterns  rather than being programmed, and it is capable of analyzing diverse data types with great flexibilities [17, 18]. ML techniques contain multiple types of classification methods, and common methods for health service research include regularized logistic regression, decision trees, neural networks (NN), and deep learning [19–21].

Recent reviews have demonstrated that ML techniques can be applied  for prediction of various types of outcomes, including disease diagnosis [22, 23], disease prognosis [24–26], or therapeutic outcomes [27, 28]. With respect to predicting readmission outcomes, very few reviews systematically gathered information of predictive models for readmission outcomes [29–33], and even fewer reviews involved the use of ML techniques for readmission outcomes [29–31]. Only three reviews involved the use of ML techniques for the readmission outcomes, but none of these reviews conducted ML method-focused evaluation of predictive models on hospital readmission [29–31]. One review specifically focused on electronic medical record (EMR) data-based readmission models between 2015 and 2019 and provided an evaluation of all such validated models.  However, this review included models based on all types of data analysis, without focused evaluation of ML techniques [29]. Another review provided an overview of predictive models for readmission until 2017 based on all types of statistical methods, including ML algorithms [30]. Christodoulou et al. specifically evaluated the use of ML for all clinical outcomes, without a focus on readmission outcomes [31]. Therefore, a gap still exists in the latest knowledge about predictive models for hospital readmission that leverages the ML techniques based on all types of databases across different healthcare settings in the US. This review focuses on predictive models of readmission that specifically use ML techniques. The objective of this scoping review was to synthesize the current literature on the types of ML techniques utilized in predicting hospital readmissions in the US. The secondary objective of this scoping review was to summarize predictive performance in terms of Area Under the Curve (AUC) across different ML algorithms for hospital readmission prediction.

## Methods

### Data sources and systematic searches

This scoping review used the Preferred Reporting Items for Systematic Reviews and Meta-Analysis Extension for Scoping Reviews (PRISMA-ScR) statement [34] to guide conduct and reporting. The Checklist for Critical Appraisal and Data Extraction for Systematic Reviews of Prediction Modelling Studies (CHARMS) [35, 36] was used to guide items to extract from the prediction models. With the assistance from an academic librarian for the Health Sciences, the authors developed the search strategies. The authors searched the databases of PUBMED, MEDLINE, and EMBASE from January 1, 2015 to December 10, 2019 to identify all potentially observational studies of applying ML techniques in hospital readmission risk prediction based on datasets of the US population. Only studies published after 2015 were included because we wanted the most recent evidence. The exact search syntax was also customized for the databases of PUBMED, MEDLINE, and EMBASE. The search syntax included search terms related to “hospital readmission” and “machine learning”. The readmission outcome refers to the readmission following any-or-all-cause index hospitalization, and ML techniques encompassed a broad range of methods; search syntax related to these terms was developed based on previous literature [19, 23, 37–39]. Searches were also limited to studies published in the English language as the review focused on the US population. The information on comprehensive search strategies and results obtained from each database are provided in Additional supporting file 1: Supporting Information Part I.

### Eligibility criteria and study selection

The initial citations and records found through database searching were imported into the COVIDENCE online software [40]. All duplicate studies were then identified and removed by the software. The titles and abstracts of these resulting articles were independently screened by two authors (Y.H. and A.T.) to identify articles that contained the concepts of ML-based hospital readmission predictive models, and any disagreement between the two authors was solved by a third reviewer (S. C.). The full text documents of these resulting articles identified as potentially relevant based on their title and abstract were retrieved, added into COVIDENCE platform, and further screened for eligibiity. Inclusion and exclusion criteria for full-text eligibility were made prior to the literature search and were in accordance with the search strategy in the identification process. The full-text articles of these potentially relevant references were evaluated for final inclusion independently by two authors (Y.H. and A.T.). Any discrepancies between the two reviewers were resolved by a third reviewer (S.C.).

Articles eligible for inclusion were as follows: (1) must use at least one ML technique for hospital readmission prediction; (2) must report details of the performance of the predictive risk model in terms of AUC; (3) the predictive risk modeling  involved US population-based databases; (4) be an original research paper; and (5) full texts in the English language. In addition, studies with the outcome of interests not relevant to hospital readmission were excluded, and studies that were randomized controlled trials (RCT), reviews, or conference abstracts were also excluded. The PRISMA flow diagram was used to guide the reporting of study identification and selection [34]. The information on comprehensive inclusion/exclusion criteria is provided in Additional supporting file 1: Supporting Information Part II.

### Data extraction

This review focused on summarizing ML techniques utilized for modeling and corresponding model performances. The list of extraction items was supported by prior literatures that involved the use of ML in readmission prediction [29–31] and was refined based on discussions among the authors. For the eligible articles included for this review, one author (Y.H.) extracted the following information, and all the information was validated by another author (A.T.). Any discrepancies between the two reviewers were resolved by a third reviewer (S.C.). The data extraction spreadsheets with extracted items involved Microsoft Excel. The extracted items were: (1) Study characteristics, including first author and publication year, data source, population and setting, sample size, outcomes studied (see Additional supporting file 2: Supporting Information Table S1); (2) Model performances, including ML-based algorithm utilized, model description, model validation, model discrimination (see Additional supporting file 2: Supporting Information Table S2); (3) Variables used as predictors in the models (see Additional supporting file 2: Supporting Information Table S3); (4) Other model performance measures, including accuracy, sensitivity, specificity, precision, recall, F1 score and method of addressing class imbalance problem (see Additional supporting file 2: Supporting Information Table S4); and (5) Quality assessment (see Additional supporting file 2: Supporting Information Table S5). All supporting information was organized based on the ML method to allow the cross-linkage between the tables. The items reported in this scoping review according to PRISMA-ScR and CHARMS guidelines can be found at Additional supporting file 3 and Additional supporting file 4, respectively.

### Quality assessment

The Quality in Prognosis Studies (QUIPS) tool was used to assess the quality of included studies [41]. This validated quality assessment tool includes six domains: study population, study attrition, prognostic factor measurement, outcome measurement, study confounding, and statistical analysis/reporting. The QUIPS tool was used to assess the quality of studies by prior studies involving modeling for readmission [30, 32] or clinical outcomes [42], and was tailored for scoping review based on prior reviews related to ML modeling for readmissions [29, 31].

From each reviewed study, the following items in each domain were elaborated in this scoping review: (1) Study population: ‘is there an adequate description of study population?’; (2) Study attrition: ‘did the study provide an adequate description of follow-up information, e.g., describing any method for handling loss-to-follow-up or deaths?’; (3) Prognostic factor measurement: ‘did the study provide an adequate description of measurement of prognostic factors, e.g., describing any imputation method for handling missing data?’; (4) Outcome measurement: ‘is there a clear definition of the readmission outcome?’; (5). Study confounding measurement and accounting: ‘did the study accounted for potential confounding factors from more than three of following domains, such as demographic factors, social determinants of health (SDoH), primary diagnosis or comorbidity index, illness severity, mental health comorbidities, overall health and functional status, prior use of medical services hospitalizations?’; and (6) Statistical analysis/reporting: ‘did the study conduct any model validation procedure?’

The ratings of ‘yes’, ‘partly, ‘no’ or ‘unclear’ were scored to each individual domain to grade the studies. The quality for each study was defined with ‘low’, ‘moderate,’ or ‘high’ based on the combined results of individual domains [42, 43]. The study was considered as ‘high’ quality if the answer was ‘yes’ or ‘partly’ for more than four domains. The study was considered as ‘moderate’ quality if more than three domains were the answer of ‘yes’ or ‘partly’. Lastly, the overall study was defined as ‘low’ quality if only two or less than two domains were provided with the answer of ‘yes’ or ‘partly’. The quality assessment was performed by two investigators (Y.H. and A.T.), and a third independent investigator (S.C.) resolved any disagreements for which consensus could not be reached by the two reviewers (Y.H. and A.T.).

### Data synthesis and analysis

Firstly, a qualitative review and synthesis of study characteristics were performed, with a focus on summarizing information on data sources, sample size, study population, and types of readmission outcomes. Secondly, a qualitative review and synthesis of model characteristics were conducted, focusing on summarizing ML algorithms utilized, model performance in terms of AUC, model validation, and use of variables. All ML techniques that were used for hospital readmission prediction in each study were comprehensively synthesized. The extracted ML algorithms were then grouped into several broad ML categories, based on our knowledge and previous literature that involved the use of ML algorithms [19, 30, 31].

Model performance was extracted in terms of AUCs of different ML models for each study (See Additional supporting file 2: Supporting Information Table S2) to further generate a comprehensive summarization of model performance by ML method. Besides AUC, this review also extracted other metrics, such as precision, and recall, which are found to be more appropriate for imbalanced datasets [44], however, the paucity of studies reporting precision or recall metrics did not permit an analysis of model performance by these metrics (See Additional supporting file 2: Supporting Information Table S4). If a study developed more than one model for the same ML algorithm (e.g., based on different predictor sets or for more than one outcome), the maximum AUC was recorded for the ease of presentation of AUC by ML method in data synthesis. In addition, AUC values in the following order of priority were used: if studies provided AUC both for training and validation datasets, only validation AUC would be reported; however, when the study was ambiguous about the datasets where the AUC was drawn from, the reported AUC was used. Based on the extracted data, the AUCs of different types of ML algorithms were visually presented in boxplot and beeswarm plot stratified by the ML category. On further analysis, the AUCs by different ML categories were summarized in descriptive statistics, including estimates of mean, median, range, Interquartile range (IQR), standard deviation (SD). The data visualization plotting and analysis of AUCs calculation were done by R software [45].

## Results

**Fig. 1:**
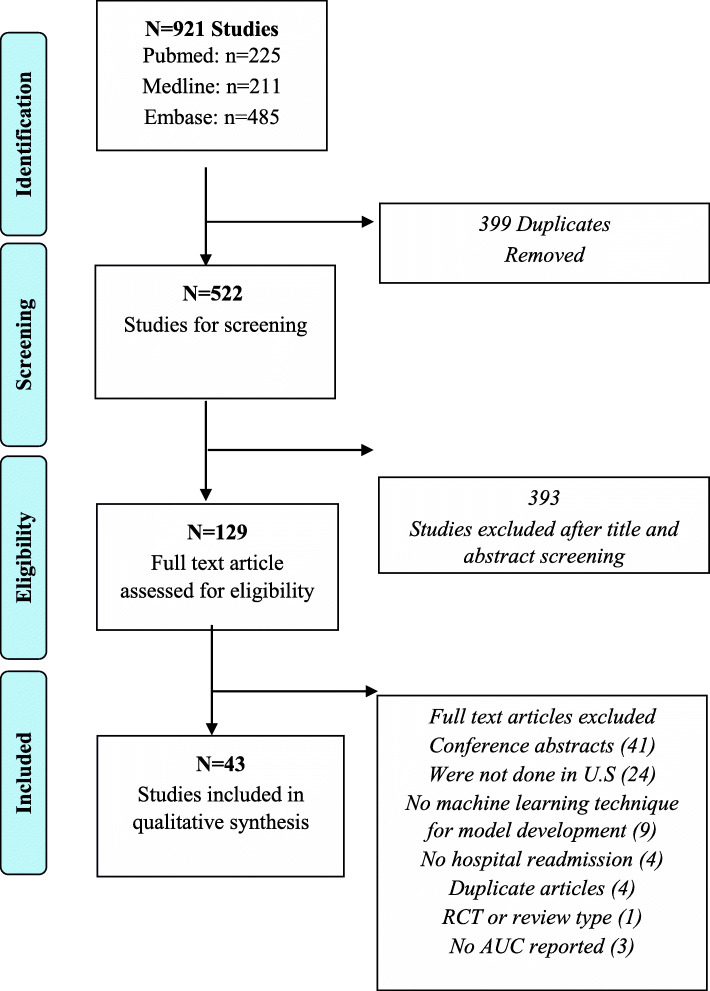
Flow diagram for study selection

Among 921 studies identified, the titles and abstracts of 522 unique papers were screened after removing duplicates. After excluding 393 records, the remaining 129 resulting citations in full-text form were assessed for full-text eligibility. A total number of 43 studies that met our inclusion criteria were identified in this scoping review (Fig. 1 [34]). The characteristics of these included studies are listed in detail in Additional supporting file 2: Supporting Information Table S1.

Readmission risk prediction involved a variety of ML techniques. Some studies used traditional statistical modeling such as logistic regression [46–48], generalized linear model (GLM) [49], Poisson regression [50], or other previously published algorithms for predicting hospital readmissions, such as Stability and Workload Index for Transfer (SWIFT) score and Modified Early Warning Score (MEWS) scores for Intensive Care Unit (ICU) readmission risk [51], CMS risk prediction model for Inpatient Rehabilitation Facilities (IRF) readmission rate [52] and current standard LACE score or Hospital Scores for hospital readmission rate [53]. Given the review focused on ML-based predictive models, only extracted information about ML-based algorithms was utilized; additionally, if traditional modeling methods, such as logistic regression, were applied with the ML strategy, such as regularization for variable selection, then they were included and grouped as “regularized logistic regression” for further evaluation. The details of model characteristics, other reported model performances, and the use of variables are summarized in Additional supporting file 2: Supporting Information Table S2-S4, respectively.

### Characteristics of the selected studies

#### Data sources and sample size

Forty-two (98%) studies clearly specified the type of data utilized for model development, and one study did not mention the type of data (2%). The majority of studies were based on EMR data (24, 56%). Studies utilized  a single hospital-based EMR (10, 26%), multiple hospitals in a single region or affiliated within the same health system (11, 26%), and national wide hospital data (2, 5%). Another common data source was population-based data sources (15, 35%) from payers, national surveys, and direct study of patients, including Medicare database (4, 9%), national surveys (American College of Surgeons National Surgical Quality Improvement Program (ACS NSQIP) (4, 9%), US renal data system (1, 2%), Healthcare Cost and Utilization Project (HCUP) (3, 7%)), patient registry (2, 5%), and randomized controlled trial datasets (1, 2%). The remaining four studies utilized administrative claims data (4, 9%), with studies utilizing a health system administrative claims (3, 7%) and one study utilizing administrative claims cross-matched to EHR data (1, 2%). The median total sample size was 23,882 (range: 132–594,751).

#### Study population and readmission outcomes

The readmission outcome was binary in all the studies that were included in this review. A total of 42 studies (98%) clearly specified the type of readmission outcomes with detailed information about the definition for readmission outcomes to be predicted in the study, and only one study (2%) did not clearly mention the type of readmissions outcome [54]. The majority of studies considered only one type of readmission rate (39, 91%), while other studies (4, 9%) used more than one readmission rate. A majority of studies used 30-day readmission (36, 84%), among which some studies were focusing on unplanned or unpreventable readmission (7, 16%), while other studies (7, 16%) used  other outcome measures, including 60-day readmission, 90-day readmission, and 1 year-readmission.

#### Use of variables

The number and type of predictors differed across different studies. For comparative reasons, variables were categorized into the following domains: demographic factors, social determinants of health (SDoH), primary diagnosis or comorbidity index, illness severity, mental health comorbidities, overall health and functional status, prior use of medical services hospitalizations, based on previous literature that involved hospital prediction models [32]. Only one study had considered all these above domains, and about half of these studies (21, 49%) had considered variables of more than four above domains. All these studies considered demographic characteristics and primary diagnosis or comorbidity index as input predictors, and the majority of these studies considered variables of pre-index utilization (40, 93%). More than half of these studies had considered SDoH (26, 60%) and illness severity (23, 53%). Some studies had considered mental health comorbidities (12, 28%), and a few studies had considered overall health status and functional status (10, 23%).

### Model characteristics

#### Use of ML

Various ML techniques were utilized in these selected 43 studies. Most studies (25, 58%) have applied more than one ML technique, and the details of all these ML techniques are summarized in Table 1. The most popular algorithm was tree-based methods (23, 53%), including decision trees (DT) [46, 52, 54–60], random forests (RF) [48–50, 59–71] and boosted tree methods [47, 49–51, 53, 54, 59, 64–67, 71–77] (e.g. gradient descent boosting, XGboost, adaboost). The second most popular algorithm was NN (14, 33%): many studies used multiple hidden layers based deep learning techniques [60, 69–71, 77, 79, 80, 85–87] (e.g., recurrent NN, convolutional NN, deep NN, and ensemble of DL networks), while a few other studies either used one hidden layer [58, 60, 68] or did not specify the number of layers [49, 66]. Regularized logistic regression (12, 28%), including Least Absolute Shrinkage and Selection Operator (LASSO) regression [53, 64, 65, 67, 70, 71, 78–80] (L1 regularization), ridge regression [64, 70, 71, 80] (L2 regularization) and elastic-net [49, 72, 81]were third most used ML algorithm, followed by Support Vector Machine (SVM) [54, 60, 63, 65, 66, 70, 71, 82–84] (10, 23%). The other less commonly used ML algorithms included naïve Bayes network [49, 54, 70, 84], K-Nearest Neighbors (KNN) algorithm [54, 65], ensemble of methods [50, 67, 84], ,and Bayesian Model averaging [49].

**Table 1:** ML algorithms used in the studies and corresponding featuring studies. (*N* = 43 studies)

Type of ML Algorithms	Number of Studies^f^ (Percent)	Featuring Studies
**Tree-based methods**	**23 (53%)**	
*Decision Tree*	*9*	[46, 52, 54–60]
*Random Forest*	*16*	[48–50, 59–71]
*Boosted tree methods*^a^	*18*	[47, 49–51, 53, 54, 59, 64–67, 71–77]
**Regularized Logistic Regression (penalized method)**	**12 (28%)**	
*Lasso (L1 regularization)*	*9*	[53, 64, 65, 67, 70, 71, 78–80]
*Ridge Regression (L2 regularization)*	*4*	[64, 70, 71, 80]
*Elastic-Net*	*3*	[49, 72, 81]
**Support Vector Machine**	**10 (23%)**	[54, 60, 63, 65, 66, 70, 71, 82–84]
**Neural Networks**	**14 (33%)**	
NN (with multiple hidden layers, e.g. deep learning)^b^	10	[60, 69–71, 77, 79, 80, 85–87]
*CNN*	*3*	[60, 71, 80]
*RNN*	*5*	[70, 71, 79, 80, 86]
*Deep stacking network*	*1*	[69]
*Deep neural networks*	*2*	[77, 85]
*Ensemble of DL methods*	*1*	[87]
NN (with a single or unclear number of hidden layers, or unclear)	5	[58]^d^, [60]^d^, [68]^d^, [49]^e^, [66]^e^
**Other algorithms**	**10 (23%)**	
*Naïve Bayes network*	*4*	[49, 54, 70, 84]
*KNN*	*2*	[54, 65]
*Ensemble of methods*^c^	*3*	[50, 67, 84]
*Bayesian Model Averaging*	*1*	[49]

*Abbreviations*: *ML* machine learning, *Lasso* least absolute shrinkage and selection operator, *NN* neural networks, *CNN* convolutional neural network, *RNN* recurrent neural network, *DL* deep learning, *KNN* The k-nearest neighbors. ^a^it includes adaboost, gradient boosting, gradient descent boosting, boosting, XGBoost; ^b^it includes CNN, RNN, DNN, deep stacking networks, and ensemble of DL methods; ^c^DT ensembled with SVM, RF combined with SVM, tree-augmented naïve Bayesian network; ^d^one hidden layers; ^e^did not specify number of layers

^f^Since most studies have applied more than 1 machine learning algorithms, therefore the sum of the number of studies by machine learning method is greater than 43

#### Model performance

**Fig. 2:**
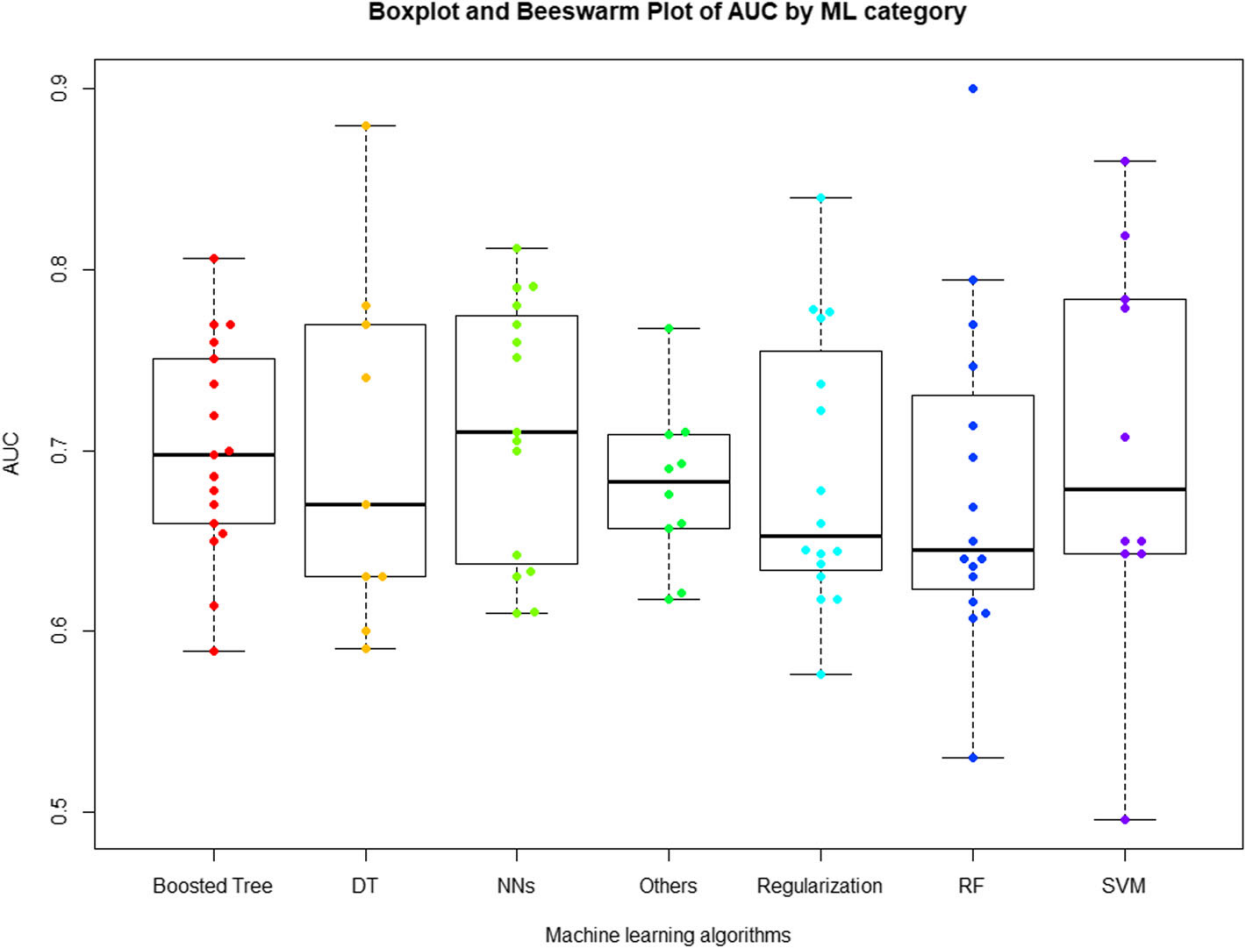
Boxplot and Beeswarm plot of AUC by ML category. Abbreviations: ML: machine learning; NNs: neural networks; RF: random forest, DT: decision tree; SVM: support vector machine

The majority of these studies (28, 65%) reported ML algorithms with AUC above 0.70, which is an indication of modest to high discrimination ability. Figure 2 showed the boxplot and beeswarm plot of AUC stratified by ML techniques. Table 2 showed the descriptive statistics of AUC by ML category. There was a range of variability within AUC reported by these studies with an average of 0.69 (0.08) and a median of 0.68 (IQR: 0.64–0.76; range: 0.50–0.90). The mean value of AUC for NN, boosted tree algorithms, random forest, decision tree, regularized logistic regression, SVM and other ML algorithms was 0.71(0.07), 0.70 (0.06), 0.68 (0.09), 0.70 (0.10), 0.69 (0.08), 0.70 (0.11), and 0.68 (0.04), respectively. The median AUC for NN algorithms was 0.71 (IQR: 0.64–0.78; range: 0.61–0.81). Median AUC was 0.70 (IQR: 0.66–0.75; range: 0.59–0.81), 0.64 (IQR: 0.63–0.72; range: 0.53–0.90), and 0.67 (IQR: 0.63–0.77; range: 0.59–0.88) for boosted tree algorithms, random forest and decision tree, respectively. The median AUC for regularized logistic regression, SVM, and other ML algorithms was 0.65 (IQR 0.64–0.75; range: 0.58–0.84), 0.68 (IQR: 0.65–0.78; range: 0.5–0.86), and 0.68 (IQR: 0.66–0.71; range 0.62–0.77), respectively.

**Table 2:** Descriptive statistics of AUC by ML category

ML category	Number of Studies^a^	Mean (STD)	Median	Min	Max	IQR
**NN**	15	0.71 (0.07)	0.71	0.61	0.81	0.64–0.78
**Boosted tree**^a^	17	0.70 (0.06)	0.7	0.59	0.81	0.66–0.75
**RF**	16	0.68 (0.09)	0.64	0.53	0.9	0.63–0.72
**DT**	9	0.70 (0.10)	0.67	0.59	0.88	0.63–0.77
**Regularized Logistic Regression**^b^	16	0.69 (0.08)	0.65	0.58	0.84	0.64–0.75
**SVM**	10	0.70 (0.11)	0.68	0.5	0.86	0.65–0.78
**Other ML algorithms**^c^	10	0.68 (0.04)	0.68	0.62	0.77	0.66–0.71

*Abbreviations*: *ML* machine learning, *NNs* neural networks, *RF* random forest, *DT* decision tree, *SVM* support vector machine, *STD* standard deviation, *IQR* the interquartile range. ^a^the total number of studies is larger than total number of included studies, because some studies used more than 1 ML algorithms. ^a^It includes adaboost, gradient boosting, gradient descent boosting, boosting, XGBoost; ^b^It includes Lasso (L1 regularization), ridge regression (L2 regularization), and elastic-net algorithms; ^c^It includes: DT ensembled with SVM, RF combined with SVM, tree-augmented naïve Bayesian network

In addition to AUC as a performance measure as specified by the current review criteria, almost half of the selected studies reported performance measure in terms of accuracy (19, 44%), while other commonly used performance measures in these selected studies, including sensitivity (22, 51%) and specificity (21, 49%). A small number of studies reported precision (5, 12%), recall (5, 12%), or F1 score (5, 12%). Only a few studies reported the methods to address imbalanced data (7, 16%).

#### Model validations

Thirty-seven studies (86%) applied some method for validation, and six studies did not use any type of validation method (14%). Table 3 showed the model validation methods among these included studies. The details of the types of model validation used in each study could be found in Additional supporting file 2: Supporting Information Table S2. Twenty-one studies (49%) randomly partitioned data into training/testing parts or training/validation/testing parts [51, 53, 55, 56, 58, 60, 65–67, 70, 73, 74, 76, 78, 79, 82–87], and most of these studies utilized some form of cross-validation in the training sets for model construction. Thirteen studies (30%) validated using various types of resampling procedures, such as k-fold cross-validation [49, 54, 57, 61, 68, 69, 77, 81] (19), stratified k-fold cross-validation [61, 80], repeated k-fold cross-validation [48], and repeated random test-train splits [50]. Only four studies (9%) used some form of external validation methods, including splitting training/test datasets by time [48, 59, 63], or used separate independent data for validation [57].

**Table 3:** Overview of methods for model validation across studies (*N* = 43)

Type of validation	Number of studies (Percent)	Featuring studies
**Internal validation**	**33 (77%)**	
**Training/testing split**	**21 (49%)**	[51, 53, 55, 56, 58, 60, 65–67, 70, 73, 74, 76, 78, 79, 82–87]
**Resampling**	**12 (28%)**	
*k-fold cross-validation*	*8*	[49, 54, 57, 61, 68, 69, 77, 81]
*Stratified k-fold cross-validation*	*2*	[61, 80]
*Repeated k-fold cross-validation*	*1*	[48]
*Repeated random test-train splits*	*1*	[50]
**External validation**	**4 (9%)**	
*Split by time*	*3*	[48, 59, 63]
*Separate dataset*	*1*	[57]
**No Validation**	**6 (14%)**	[46, 47, 52, 62, 72, 88]

### Quality assessment

Most studies (37, 86%) were of high quality based on the appraisal of six domains of the QUIPS tool. A few studies failed to report how to handle a loss to follow-up issues (such as deaths or other reasons causing the missing values). Many studies did not provide an adequate definition of outcome measures (such as inclusion or exclusion criteria). The full description of quality assessment for all included studies is summarized in Additional supporting file 2: Supporting Information Table S5.

## Discussion

In this scoping review, 43 studies involving ML prediction models for hospital readmission were evaluated. These models were developed and tested in a variety of settings and populations in the US using health care data from insurance claims, EMRs, or surveys. Tree-based methods, NN, and regularized logistic regression were the most popular ML approaches used to predict readmission risk. There was variation in model performance in terms of AUC across these prediction models. Most of the studies have applied multiple methods for validation. Domains of variables, including sociodemographic factors, SDoH, primary diagnosis or comorbidity index, illness severity, comorbidities, overall health, and functional status, were generally included for the development of ML prediction models. The overall quality in most of these studies was high.

To our knowledge, this is the first review to provide a focused evaluation of ML models for readmission risk prediction. This scoping review suggests growing importance of ML methods for a variety of medical outcomes. In recent years, ML is increasingly used to predict a wide range of clinically relevant outcomes with the availability of health data, including cancer [25] or dementia [24] prognosis, neurosurgical outcomes [26, 89], and clinical diagnostic outcomes [31]. The value of ML in the readmission risk prediction has not been systematically investigated, given the importance of readmissions as a quality indicator. Hence, this review offers needed insights on the cutting-edge applications of ML methods for readmission risk prediction. The findings of this review are consistent with other reviews indicating the popularity of tree-based methods and NN in predicting hospital readmissions [29, 30]. Mahmoudi et al., limited to EMR data sourced studies, found that the random forest and NN as the most popular ML methods for predicting readmission [29]. Artetxe et al. also found that tree-based methods and SVM were the most utilized ML algorithms for predicting readmission outcomes [30]. While these two reviews examined both traditional regression and ML models, this review specifically evaluated ML predictive models for hospital readmission and associated model parameters, data sources, and others to provide contemporary empirical evidence on the applications of ML techniques.

In this review, the performance of ML methods varied. The NN and boosted tree algorithms generally performed  better based on the C-statistic. These observations are in alignment with the existing body of literature showing that the strong performance of boosted tree algorithms and NN algorithms for readmission risk prediction [29–31]. The NN performed well in other clinical outcome predictions as it recognizes the patterns of data through labeling/clustering of raw input data and applying layers of neuron-like processing units [90, 91]. The boosted tree algorithm is an ensemble method for regression and classification problems by combining the strengths of regression trees and boosting, and it builds the prediction model in an adaptive and iterative fashion [92, 93]. Besides C statistics, most of the reviewed models did not report other measures, such as precision or recall, nor discussed the methods to address imbalanced data. This finding highlighted the need for a complete reporting on a comprehensive list of metrics for model evaluation to enable an insightful comparison of model performance by ML methods. A study comparing strategies for addressing class-imbalance problems is also needed, so that future researchers may benefit from addressing imbalanced outcomes for readmission prediction.

The variability of AUC across these evaluated models should be considered in light of factors influencing the predictive performances, including types of ML classification methods, predictors included for modeling, and selection of validation methods. Firstly, more complex models, such as deep learning methods, namely NNs with multiple layers, are considered to have the greatest potential to boost predictive performance [60, 68–70, 77, 79, 85], and often dominated comparative models with other ML algorithms [60, 66, 68–70, 77, 79, 85]. However, these sophisticated modeling approaches, especially deep learning models, involve a time-consuming process of parameter tuning and are difficult for interpretion. Secondly, one challenge with achieving a model with high performance for the readmission outcome is to have an inclusion of rich information of varieties of predictors, given the multidimensional nature of readmission problem [94, 95] and the dependence of the performance of ML techniques on the quality and information of input data [96–98]. This review noted the absence of studies that incorporated variables in the domains of overall health and function or mental health comorbidities, and such problems have been identified by prior reviews [29, 32]. This review also found the improvement in prediction ability offered by models aided with natural language processing techniques that are able to extract unstructured information, such as topic features or frequently used words from clinical notes and/or discharge dummies [71, 77, 83, 86, 87]. Future studies should include a comprehensive list of factors to study readmission problem.  Innovation in analyzing unstructured data can also help to collect relevent variables types for inclusion. Furthermore, different types of validation methods were conducted for the ML models, and most models  involved internal validation. The problem of lack of standardized validation methods [99] and absence of external validation using independent datasets [23, 28] in ML studies has been noted by other reviews. The external validation of these predictive models might increase the model generalizability. More importantly, the frameworks for ML model development, including standardized validation procedures, are needed to facilitate the implementation of ML for predicting readmissions and other clinical problems.

This review synthesized and evaluated predictive models for hospital readmissions in the US that leverage the ML techniques. This review has several strengths. Firstly, this review concurs with a body of existing literature indicating the growing use of ML approaches for clinical risk prediction problems and advances the evidence on the common ML methods designed specifically to address the readmission risk prediction problem. Given the limited and emerging body of ML-related literature on readmission predictive modeling, this review is the first attempt to conduct a focused synthesis of the literature on ML approaches for predicting readmission outcomes. Secondly, the review included a list of evaluation metrics to assess the model performances of the ML models and were able to generate some insights on the performances of these ML methods in predicting readmission outcomes. In addition, this review gathered some important parameters involved in the ML model development, including data sources and validation methods. This review was performed in accordance with two guidelines: the PRISMA-ScR checklist and the CHARMS guidelines for consistency and transparency. Most of the included studies were of high quality and thus ensuring that the internal validity of the finding in this scoping review is high.

This review has several limitations. Firstly, this review focused on the ML approaches used for predicting readmission outcomes and did not summarize the most predictive features for readmission risk. This is a limitation because understanding significant contributing variables driving readmission risk might be useful for clinicians in making actionable care plans for readmission reduction. Secondly, in order to provide a comprehensive summary of the latest ML methods for building readmission risk prediction, studies were not limited by diagnosis within the population and therefore cannot comment on the performance of ML methods for readmission prediction among a specific disease population. Given readmission problem is disease-specific, future studies should further evaluate the relative value of different ML approaches in assessing disease-specific readmission outcomes. Furthermore, this review did not investigate which factors influence the difference in performance within each ML method. These factors are dependent on the particular application of the ML method in question, and such factors should be best analyzed by comparing different scenarios on the same data sets. Several limitations should also be noted among studies developing these predictive models: as discussed above, most of the reviewed studies did not report other metrics besides AUC; therefore, the performance of these predictive models based on such measures was not evaluated. Also, most validations were done internally, and this limits their generalizability to a new setting. Lastly, this review specifically focused on ML approaches for readmission outcome prediction and therefore cannot comment on the performance of traditional statistical methods. Future comparative studies on the performance between these traditional statistic methods with ML methods could guide identifying the method with optimal performance in readmission risk prediction.

Overall, this review provides promising support for ML for the development of advanced risk prediction models for readmission in the US population. Comparison of ML readmission risk modeling methods in terms of performance should be considered in light of the unique characteristics of each study and model performance parameters. The benefits of developing ML models for predicting readmission in clinical settings will continue to increase with the inclusion of additional clinical measures from unstructured data and the implementation of standardized validation methods. Future research should focus more on identifying which algorithms have optimal performance for readmission prediction and studying the model development framework to optimize relevant ML algorithms for predicting readmission risk.

## Conclusions

The current review found that various types of ML techniques have been utilized in hospital readmission prediction with tree-based methods, NN, regularized logistic regression, and SVM as the most commonly used algorithms. There is also a variation of model performance in terms of AUC among these algorithms, and the performance of these ML models varied due to various reasons. The boosted tree algorithms and NN algorithms were often used and had a strong model performance. Inclusion of variables across all domains and performing external validation could allow for improved model performance and reliability. These findings have implications for leveraging the ML methods for assessing readmission risk. Continued efforts could be focused on optimizing the performance of ML algorithms to predict hospital readmissions and developing frameworks for ML model building to integrate these models into clinical operations with a goal to improve quality of care and reduce health care costs.

## Supplementary Information

**Additional file 1: **Search Strategy and Statement of Questions with Reference to PICOS. This file includes Part I and Part II. **Part I**, Full Electronic Search Strategies for PUBMED, MEDLINE and EMBASE Databases and Results. This file includes the search terms used in the above databases. **Part II**, Inclusion/Exclusion criteria for screening articles. (e.g. PICOS, timing, setting)

**Additional file 2: **Extracted Items for Included Studies. This file includes Table S1-Table S5. **Table S1.** Information about study characteristics, including first author and publication year, data source, population and setting, sample size, and outcome studied. **Table S2.** Information about model performances, including ML-based algorithm utilized, model description, model validation, and model discrimination. **Table S3.** Information about variables used as predictors in the models. **Table S4.** Information about other model performance measures, including accuracy, sensitivity, specificity, precision, recall, or F1 score, and method of addressing class imbalance problem. **Table S5.** Information about quality assessment

**Additional file 3: **Reporting of PRISMA-ScR Checklist. This file includes Table S1. **Table S1.** Reporting of PRISMA-ScR Checklist

**Additional file 4: **Reporting of CHARMS Checklist. This file includes Table S1. **Table S1.** Reporting of CHARMS Checklist

## Data Availability

The corresponding author can provide the material used and data analyzed on request.

## Abbreviations

CMS: Centers for Medicare and Medicaid Services
ML: Machine Learning
NN: Neural networks
AUC: Area Under the Curve
RCT: Randomized clinical trial
PRISMA-ScR: the Preferred Reporting Items for Systematic Reviews and Meta-Analysis Extension for Scoping Reviews
CHARMS: CHecklist for critical Appraisal and data extraction for systematic Reviews of prediction Modelling Studies
QUIPS: The Quality in Prognosis Studies
IQR: Interquartile range
GLM: Generalized linear model
SWIFT: Stability and Workload Index for Transfer
MEWS: Modified Early Warning Score
ICU: Intensive Care Unit
IRF: Inpatient Rehabilitation Facilities
SDoH: Social determinants of health
SVM: Support vector machine
KNN: K-nearest neighbor algorithm
EMR: Electronic medical record
DT: Decision tree
RF: Random forest
ROB: Risk of bias
CNN: Convolutional neural network
RNN: Recurrent neural network
DL: Deep learning
