# Supplementary material for: Application of machine learning in predicting hospital readmissions: a scoping review of the literature
Source: BMC Med Res Methodol. 2021 May 6;21:96. doi: 10.1186/s12874-021-01284-z (PMC8101040; doi:10.1186/s12874-021-01284-z)
Supplement: Supplementary file 1 — Additional file 1: Search Strategy and Statement of Questions with Reference to PICOS. This file includes Part I and Part II. Part I, Full Electronic Search Strategies for PUBMED, MEDLINE and EMBASE Databases and Results. This file includes the search terms used in the above databases. Part II, Inclusion/Exclusion criteria for screening articles. (e.g. PICOS, timing, setting) [file 12874_2021_1284_MOESM1_ESM.docx]

**Additional Supporting File 1: Part 1, Full Search Strategy**

**Databases Searched**

| Date | Name | Records |
| --- | --- | --- |
| 12/10/2019 | PUBMED | 225 |
| 12/10/2019 | MEDLINE EBSCO | 211 |
| 12/10/2019 | EMBASE Ovid | 485 |

| **Database: PUBMED**  **Search Date: December 10th 2019** | | |
| --- | --- | --- |
| 1 | "readmi*"[Title/Abstract] OR "rehosp*"[Title/Abstract] OR "re admi*"[Title/Abstract] OR "re hosp*"[Title/Abstract] OR "postdischarge*"[Title/Abstract] OR "post discharge"[Title/Abstract] | 45,829 |
| 2 | patient readmission[MeSH Terms] | 15,973 |
| 3 | 1 or 2 | 49,700 |
| 4 | “machine learning”[Title/Abstract] OR “artificial intelligence”[Title/Abstract] OR “supervised learning”[Title/Abstract] OR “Unsupervised machine learning”[Title/Abstract] OR “Reinforcement Learning”[Title/Abstract] OR “pattern recognition*”[Title/Abstract] OR “Pattern classification”[Title/Abstract] OR “Computational Intelligence*”[Title/Abstract] OR “computer reasoning”[Title/Abstract] OR “Machine Intelligence”[Title/Abstract] OR “Knowledge Representation*”[Title/Abstract] OR “deep learning”[Title/Abstract] OR “support vector*”[Title/Abstract] OR “Neural net*”[Title/Abstract] OR “Nearest Neighbo*”[Title/Abstract] OR “Naive Bayes”[Title/Abstract] OR “bayesian “machine learning”[Title/Abstract] OR “artificial intelligence”[Title/Abstract] OR “supervised learning”[Title/Abstract] OR “Unsupervised machine learning”[Title/Abstract] learning”[Title/Abstract] OR “Bayesian belief network*”[Title/Abstract] OR “Bayesian net*”[Title/Abstract] OR “Decision Tree*”[Title/Abstract] OR “regression tree*”[Title/Abstract] OR “classification tree*”[Title/Abstract] OR “Random forest”[Title/Abstract] OR “Regularized logistic”[Title/Abstract] OR “Linear Discriminant Analysis”[Title/Abstract] OR “language processing”[Title/Abstract] OR “text analysis”[Title/Abstract] OR “Gaussian process”[Title/Abstract] OR “Least Absolute shrinkage selection operator”[Title/Abstract] OR “LASSO”[Title/Abstract] OR “elastic net”[Title/Abstract] OR “relevance vector machine”[Title/Abstract] OR “boosting”[Title/Abstract] OR “extreme learning machines”[Title/Abstract] OR “association mining”[Title/Abstract] OR “association rule*”[Title/Abstract] OR “analogy”[Title/Abstract] OR “case based reasoning”[Title/Abstract] OR “Long Short Term Memory”[Title/Abstract] OR ”genetic algorithm*”[Title/Abstract] | 167,715 |
| 5 | machine learning[MeSH Terms] | 14,551 |
| 6 | 4 or 5 | 170,027 |
| 7 | #3 and #6 | 289 |
| 8 | English  2015-Current | 225 |

| **Database: MEDLINE EBSCO**  **Search Date: December 10th 2019** | | |
| --- | --- | --- |
| 1 | readmit* or readmission* or rehospital* or “re admit*” or “re admission*” or “re hospital*” or postdischarge* or “post discharge” | 43,043 |
| 2 | MH "Patient Readmission" | 15,959 |
| 3 | #1 or #2 | 46,962 |
| 4 | “machine learning” OR “artificial intelligence” OR “supervised learning” OR “Unsupervised machine learning” OR “Reinforcement Learning” OR “pattern recognition*” OR “Pattern classification” OR “Computational Intelligence*” OR “computer reasoning” OR “Machine Intelligence” OR “Knowledge Representation*” OR “deep learning” OR “support vector*” OR “Neural net*” OR “Nearest Neighbo*” OR “Naive Bayes” OR “bayesian learning” OR “Bayesian belief network*” OR “Bayesian net*” OR “Decision Tree*” OR “regression tree*” OR “classification tree*” OR “Random forest” OR “Regularized logistic” OR “Linear Discriminant Analysis” OR “language processing” OR “text analysis” OR “Gaussian process” OR “Least Absolute shrinkage selection operator” OR “LASSO” OR “elastic net” OR “relevance vector machine” OR “boosting” OR “extreme learning machines” OR “association mining” OR “association rule*” OR “analogy” OR “case based reasoning” OR “Long Short Term Memory” OR ”genetic algorithm*” | 162,154 |
| 5 | (MH "Machine Learning+") OR (MH "Supervised Machine Learning+") OR (MH "Unsupervised Machine Learning") | 14,522 |
| 6 | #4 or #5 | 162,157 |
| 7 | #3 and #6 | 268 |
|  | Limiters:  Date of Publication: 20150101-20191231;  English Language; | 211 |

| **Database: EMBASE Ovid**  **Search Date: December 10th 2019** | | |
| --- | --- | --- |
| 1 | (readmi* or rehosp* or 're admi*' or 're hosp*' or postdischarge* or 'post discharge').mp. | 95,759 |
| 2 | exp hospital readmission/ | 58,534 |
| 3 | 1 or 2 | 95,759 |
| 4 | ('machine learning' or 'artificial intelligence' or 'supervised learning' or 'Unsupervised learning' or 'Reinforcement Learning' or 'pattern recognition*' or 'Pattern classification' or 'Computational Intelligence*' or 'computer reasoning' or 'Machine Intelligence' or 'Knowledge Representation*' or 'deep learning' or 'support vector*' or 'Neural net*' or 'Nearest Neighbo*' or 'Naive Bayes' or 'bayesian learning' or 'Bayesian belief network*' or 'Bayesian net*' or 'Decision Tree*' or 'regression tree*' or 'classification tree*' or 'Random forest' or 'Regularized logistic' or 'Linear Discriminant Analysis' or 'language processing' or 'text analysis' or 'Gaussian process' or 'Least Absolute Shrinkage Selection Operator' or 'LASSO' or 'elastic net' or 'relevance vector machine' or 'boosting' or 'extreme learning machines' or 'association mining' or 'association rule*' or 'analogy' or 'case based reasoning' or 'Long Short Term Memory' or 'genetic algorithm*').mp. | 281, 242 |
| 5 | \|  \| exp machine learning/ or exp artificial intelligence/ \| \| --- \| --- \| | 180,997 |
| 6 | 4 or 5 | 346,690 |
| 7 | 3 AND 6 | 662 |
|  | limit 6 to (English language year="2015 -Current") | 485 |

**Additional Supporting File 1: Part II. Inclusion/Exclusion Criteria for Screening Articles**

*Population:* Patients admitted to any facility that are accessible for patients’ admissions, including inpatient facility, emergency rooms, psychiatry or other settings.

*Intervention:* Development of a predictive model of hospital readmission utilizing any type of machine learning techniques.

*Comparator:* None.

*Outcomes:* hospital readmission.

Timing: Between January 1, 2015 and December 31, 2019.

Setting: Exclude studies that were conducted in health systems of countries other than U.S.

*Inclusion Criteria:*

(1). must use at least one machine learning technique for hospital readmission prediction; (2). must report details of the performance of the risk predictive model in terms of AUC (2). the risk predictive modelling is among U.S. population-based databases; (3). be an original research paper; (4). full texts in English. Study were excluded if:

*Exclusion Criteria:*

(1). articles did not use any type of machine learning techniques for risk prediction; (2). study outcome of interests not relevant to hospital readmission; (3). the databases applied were not among U.S. population (4). it was RCT or review type, or conference abstract; (5). it was not written in English.
