# Supplementary material for: Application of machine learning in predicting hospital readmissions: a scoping review of the literature
Source: BMC Med Res Methodol. 2021 May 6;21:96. doi: 10.1186/s12874-021-01284-z (PMC8101040; doi:10.1186/s12874-021-01284-z)
Supplement: Supplementary file 2 — Additional file 2: Extracted Items for Included Studies. This file includes Table S1-Table S5. Table S1. Information about study characteristics, including first author and publication year, data source, population and setting, sample size, and outcome studied. Table S2. Information about model performances, including ML-based algorithm utilized, model description, model validation, and model discrimination. Table S3. Information about variables used as predictors in the models. Table S4. Information about other model performance measures, including accuracy, sensitivity, specificity, precision, recall, or F1 score, and method of addressing class imbalance problem. Table S5. Information about quality assessment [file 12874_2021_1284_MOESM2_ESM.docx]

**Additional file 2: Part II.Table S1-Table S2**

**Table S1. Characteristics of Included Studies on Hospital Readmission Predictive Models (N=43 studies)**

| **Study** | **Data Source** | **Population and Setting** | **Sample Size** | **Type of Readmission Measures** | **Actual Readmission Rate, %** |
| --- | --- | --- | --- | --- | --- |
| Kang et al, 2016 | Medicare Outcome and Assessment Information Set (OASIS-C) | HF patients receiving tele-homecare discharged from an in-patient facility in 2010 | 552 patients | 60-day rehospitalization | 35.87% |
| Brom et al, 2019 | A level 1 trauma center based electronic health records (EHR) | All adult patients discharged from a trauma center in urban area from 08/2017-10/2017 | 2,165 patients | 30-day unplanned readmission | 11.20% |
| Yeo et al, 2016 | American College of Surgeons National Surgical Quality Improvement Program (ACS NSQIP) database | Elderly adults above 65 who underwent surgery for colorectal cancer from multiple US hospitals from 2011-2012 | 2,117 patients | 30 days readmission | 10% |
| Edgcomb et al, 2019 | University of California Health Care System EHR | Adult patients with coexisting bipolar disorder and serious medical illness admitted to 2 academic medical centers within UCLA health system from 2006-2016 Comorbid Medical Illness | 552 patients | 30-day unplanned psychiatric readmission | 7-day readmission=3%; 30-day readmission=8.6%; 365-day readmission=23.5% |
| Fisher et al, 2015 | Medicare Database: functional status information from the Inpatient Rehabilitation Facility-Patient Assessment Instrument linked with Medicare Provider Analysis and Review (MedPAR) file | Patients discharged from inpatient rehabilitation from 01/2010-11/2011 | 87,577 patients | 30-Day Readmission for acute care | 25% |
| Jones et al,2019 | Medicare Outcome and Assessment Information Set (OASIS-C) | All elderly aged above 65 receiving home care within 5 days of discharge in 2012 | 43,407 patients | 30-day rehospitalization | 14.70% |
| **Study** | **Data Source** | **Population and Setting** | **Sample Size** | **Type of Readmission Measures** | **Actual Readmission Rate, %** |
| Hogan et al, 2019 | United States renal data system | Elderly patients (aged >= 65) who received a kidney transplant from multiple US hospitals from 01/2005 to 12/2014 and simultaneously enroll in Medicare | 40,461 patients | 30 days readmission; 31-90 days readmission; 91-365 days readmission | 30-day readmission=31.8%; 365 days readmission=62.9% |
| Zack et al, 2019 | Mayo Clinic Percutaneous coronary intervention registry | All patients who underwent percutaneous coronary intervention at Mayo clinic, Rochester, Minnesota, 01/2004-12/2013 | 11,709 patients with 14,349 PCI index hospitalizations | 30-day readmission for congestive heart failure related to either systolic and/or diastolic dysfunction | 0.70% |
| Mahajan et al, 2016 | Veteran Administration (VA) Health System EHR | HF patients, Veterans Affairs Palo Alto Health Care System, Palo Alto, California, for 6 years | 1,037 patients | 30-day HF readmissions | 17.36% |
| Xue et al, 2019 | a rehabilitation center within a state based large health system EHR | All patients admitted to an inpatient rehabilitation facility in Eastern North Carolina, 2001-2017 | 16,902 patients | 30 days acute care readmission | 10.00% |
| Mahajan et al, 2018 | VA Health System EHR | HF patients treated 31 hospitals from multi regions in the U.S. in 2015 | 1778 patients with 701 readmissions | 30-day HF readmissions | 39.40% |
| Kalagara et al, 2019 | ACS NSQIP database | Adult patients who had undergone lumbar laminectomy from multiple U.S. hospitals from 2011 to 2014 | 26,869 patients | 30-day rehospitalization | 5.59% |
| Merrill et al, 2018 | Nationwide Inpatient Sample and Nationwide Readmission Database | Adult patients who underwent open reduction and internal fixation (ORIF) of an ankle fracture from 2013-2014 | 33,504 patients | 30 days all-cause readmission | 5.90% |
| **Study** | **Data Source** | **Population and Setting** | **Sample Size** | **Type of Readmission Measures** | **Actual Readmission Rate, %** |
| Rojas et al, 2017 | University of Chicago Medical Center based EHR (model derivation); Beth Israel Deaconess medical center based EHR, Medical Information Mart for Intensive Care (MIMIC-III) database (model validation) | Adult ICU patients transferred to the wards at a single medical center from 11/2008-01/2016 | 18,000 patients with 24,885 ICU admissions | early ICU unplanned readmission (0-72 hours); late ICU unplanned readmission (>72 hours); ever ICU unplanned readmission | 11% |
| Chandra et al, 2019 | Administrative claims cross matched to EHR | Elderly patients (aged above 55) discharged to skilled nursing facilities from 2 Mayo Clinic hospitals, Rochester, Minnesota, 01/2009-06/2014 | 6,032 patients | 30-day readmission | 18.20% |
| Pakbin et al, 2018 | Beth Israel Deaconess medical center based EHR, Medical Information Mart for Intensive Care (MIMIC-III) database | Adult patients in intensive critical care units at Beth Israel Deaconess Medical Center, 2001 and 2012 | 46,252 patients with 58,151 index hospital admissions | ICU readmissions for 24hours,72hours, 7days, 30days | 11.69% |
| Salem et al, 2018 | an academic hospital in Houston EHR | Mental illness/adult patients with bipolar disorder comorbid medical illness admitted to psychiatric unit, an academic hospital in Houston, 07/2013-07/2018 | 714 adult patients | 30-day readmission | 8.80% |
| Rumshisky et al, 2016 | Partners HealthCare EHR | Mental illness/patients with major depressive disorder admitted to a psychiatric inpatient unit, several hospitals under a not-for-profit health care system, 1994 and 2012 | 4,687 patients with 470 readmissions | 30- day psychiatric readmission | 10.03% |
| **Study** | **Data Source** | **Population and Setting** | **Sample Size** | **Type of Readmission Measures** | **Actual Readmission Rate, %** |
| Turgeman et al, 2016 | Administrative claims of Veterans Health Administration (VHA) hospitals in Pittsburgh | CHF inpatients, VA hospitals, Pittsburgh, fiscal years 2006-2014 | 4,840 patients with 20,321 inpatient admissions | all cause readmission | 27.99% |
| Povalej et al, 2017 | Healthcare Cost and Utilization Project, State Inpatient Database for California | Morbidly obese patients, California State, 2009-2011 | 4,787 (6 records of hospitalization) -20,521 patients (with at least 2 hospitalization records) | 30-day readmission | 34.36%-43.85% |
| Mahajan et al, 2017 | VA Health System EHR | HF patients, Veterans Affairs Palo Alto Health Care System, Palo Alto, California, from 2005-2013 | 1,210 patients with 263 readmissions | 30-day HF readmissions | 23.96% |
| Nakamura et al, 2019 | Boston Children’s Hospital, Children’s Hospital Colorado, and Lucile Packard Children’s Hospital Stanford EHR | Pediatric patients (aged 29days- 18 years) admitted to regional based hospitals: Boston Children's Hospital, Children's Hospital Colorado, Lucile Packard Children's Hospital Stanford, 02/2011-02/2014 | 84,984 index admissions | 30-day readmission | 7.60% |
| Ehwerhemuepha et al, 2020 | A tertiary pediatric hospital in Southern California EHR | All pediatric inpatients (aged 29 days- 21 years), a regional based single tertiary pediatric hospital, 2013-2017 | 50,241 encounters | pediatric 7-day unpreventable readmissions | Overall=4.46%; training=4.65%; testing=4.28% |
| Tong et al, 2016 | EHR of eight Advocate Health Care hospitals in the Chicago | All inpatients, 8 hospitals (Condell Medical Center, Good Shepherd Hospital, Lutheran General Hospital, Illinois Masonic Medical Center, Good Samaritan Hospital, Christ Medical Center, Trinity Hospital and South Suburban Hospital), a large urban area, 03/2011-07/2012 | 109,421 patients with 18,707 readmissions | 30-day  all-cause non-elective readmission | 11.50% |
| **Study** | **Data Source** | **Population and Setting** | **Sample Size** | **Type of Readmission Measures** | **Actual Readmission Rate, %** |
| McKinley et al, 2018 | A WellStar Atlanta Medical Center in Atlanta EHR | HF African American Males admitted to an academic medical center, Atlanta, Georgia, 05/2012-12/2015 | 132 patients | 30-day  readmission | 28.80% |
| Kulkarni et al, 2016 | Data from a private health system | All inpatients admitted to hospitals in a private health system, during 1 year | 112,750 index admissions | 30-days unplanned readmission | 12.15% |
| Goyal et al, 2019 | ACS NSQIP | Patients undergoing cervical or lumbar spinal fusion (neurosurgery, 500 hospitals across the nation, 2012-2013 | 59,145 patients | 30 days unplanned readmission | 4.50% |
| Garcia-Acre et al, 2018 | Administrative claims of a network of 11 hospitals in Florida | Patients diagnosed with AMI, or COPD, or CHF, or PN, or DIA at 11 hospitals from Florida, 2005-2012 | 594,751 patients; AMI=11,205; CHF=9,586; COPD=7,911; DIA=6,145; PN=12,123 | 30-day preventable hospital readmissions | AMI=18%; CHF=16%; COPD=15%; DIA=12%; PD=11% |
| Mortazavi et al, 2016 | Telemonitoring to Improve Heart Failure Outcomes trial | HF patients | 1004 patients (30-day); 977 (180 day) | 30-day and 180-day all-cause; 30-day and 180-day heart failure-only readmissions | 30 d all=17.1%; 180 d all=48.9%; |
| Frizzell et al, 2017 | Get with the Guidelines Heart Failure registry (GWTG-HF) linked with Medicare Inpatient data | Elderly patients (aged above 65) admitted to GWTG-HF registry and discharged alive between 01/2005-12/2011 with enrollment in Medicare Fee-for Service A and B at discharge. | 56,477 patients | 30 days all-cause readmissions | 21.20% |
| Eckert et al, 2018 | A Madigan Army Medical Center EHR | All inpatients admitted to a tertiary care medical facility (Madigan Army Medical Center), Tacoma, Washington, 01/2014-01/2016 | 24,499 patients with 32,659 admissions (training);1,574 patients (testing); 32,219 patients with 42, 392 admissions (revised model retrospective testing) | 30 days all-cause readmissions | training=9.4%; prospective testing=9.7%; retrospective testing=12.09% |
| **Study** | **Data Source** | **Population and Setting** | **Sample Size** | **Type of Readmission Measures** | **Actual Readmission Rate, %** |
| Lodhi et al, 2017 | Four hospitals-based Nursing EHR of HANDS database | All inpatients with pain problems who had received nursing care from 4 different hospitals, 2005-2008 | 34,929 patients | readmission rate | 15% |
| Jamei et al, 2017 | Sutter Health, EHR of a large nonprofit hospital network in North California | Inpatients treated in all Sutter hospitals, excluding Skilled Nursing and other specialty facilities, in California.2009-2015, Multiple hospitals under a single health system, a single state | 335,815 patient records | 30-day hospital  readmission | 9.74% |
| Welchowski et al, 2016 | Cerner Health Facts HER | Diabetes patients, multiple hospitals, 1999-2008 | 71,518 patients | 30-day hospital readmissions | 15.88% |
| Wang et al. 2018 | Barnes-Jewish Hospital based EHR | All adult inpatients from general hospital wards (GHW) from 07/2007-07/2011; all post-surgical patients from operating room pilot data (ORP) | GHW=41,503 patient visits; ORP=700 patients | 30-day;60-day;1-year readmission | GHW: 30-d readmission=0.98%; 60-day readmission=1.30%; ORP: 30-d readmission=17.71%; 1-year readmission=17.71% |
| Hopkins et al, 2019 | ACS NSQIP | All adult patients who had undergone a posterior lumbar fusion procedure from 2011 to 2016 | 23,264 patients | 30-day readmission | 5.15% |
| Golas et al, 2018 | Partners Healthcare System based EHR | Adult HF patients, 2 Academic centers and 7 community health centers in a Massachusetts metropolitan area, 2013-2015 | 11,510 patients with 27,334 admissions | 30 day all cause readmission | 55.33% |
| **Study** | **Data Source** | **Population and Setting** | **Sample Size** | **Type of Readmission Measures** | **Actual Readmission Rate, %** |
| Reddy et al, 2018 | Cerner HealthFacts (National EHR) | Lupus inpatients, 500 healthcare facilities, 2000-2015 | 11,007 patients | 30-day readmissions | 17..2% |
| Xiao et al, 2018 | EHR& Synthetic EHR data | CHF patients (no clear setting) | EHR=5,393 patients; Synthetic EHR=3,000 patients | 30-day hospital readmissions | / |
| Lin et al, 2019 | Beth Israel Deaconess medical center based HER, Medical Information Mart for Intensive Care (MIMIC-III) database | All adults admitted to ICU of a single medical center, from 2001-2012 | 35,334 patients with 48,393 ICU stays | 30-day unplanned ICU readmission | 9.07% |
| Allam et al, 2019 | Healthcare Cost and Utilization Project, National wide Readmission Database | Adult’s HF inpatients, nationwide multiple hospitals, from 2013 onwards | 272,778 patients | 30-day all-cause readmissions | 23.60% |
| Min et al. 2019 | Geisinger Health system administrative Claims | COPD, multicenter hospitals in health system, 2004-2015 | 111,992 patients | 30-day all cause readmission | / |
| Rajkomar et al, 2018 | Health system based EHR | All adult inpatients from UCSF health system from 2012-2016, and UCM health system from 2009-2016. | 114,003 patients with 216,221 hospitalizations | 30-day unplanned readmissions | 12.90% |
| abbreviations: Medical Information Mart for Intensive Care; Medicare Outcome and Assessment Information Set, FIM: Functional Independence Measure; OASIS-C: Medicare Outcome and Assessment Information Set; ACS NSQIP: American College of Surgeons National Surgical Quality Improvement Program (ACS NSQIP) database; electronic health records (EHR); VA: Veteran Administration; HF: heart failure; UCLA: University of California, Los Angeles; ICU: Intensive care unit; AMI: Acute Myocardial Infarction; COPD: Chronic obstructive pulmonary disease; Congestive heart failure (CHF); PN: Pneumonia; DIA: Type 2 diabetes; GWTG-HF: Get With the Guidelines Heart Failure registry; UCM: University of Chicago Medicine; UCSF: University of California, San Francisco | | | | | |

**Table S2. Performances of Included Studies on Hospital Readmission Predictive Models Using ML Algorithms (N=43 Studies)**

| **Study** | **ML based Algorithms** | **Model Description (number of models, number of candidate variables, time)** | **Model Validation** | **Model Discrimination** |
| --- | --- | --- | --- | --- |
| Kang et al, 2016 | Decision tree | A model containing 85 variables (including patient's overall health status, living situation, severe pain experience, presence of skin issues, ability to dress the lower body) at the start of the homecare | training/  testing:67%/33%; 10-fold cross-validation | 0.59 |
| Brom et al, 2019 | CART | A model containing 14 variables (from domains of patient’s demographic, social characteristics, utilization patterns, Elixhauser cormobidities) available at time of discharge | training/  validation/  testing: 50%/25%/25%; cross- validation | 0.74 |
| Yeo et al, 2016 | CART | a model containing 28 variables (demographic characteristics, nutritional status, functional status, behavioral variables, and comorbidities) at the time of admission | / | 0.63(0.59–0.67) |
| Edgcomb et al, 2019 | CART | two models trained separately by unbalanced and balanced trees containing 249 variables (demographics, medical and psychiatric diagnoses, medication regimen and disposition, time-varying predictors (number of encounters with a given chief complaint, the number of hospitalizations, ambulatory visits, ED vistis and procedures; modal pain score; financial charges), psychiatric and medical comorbidities, ECI) | 10-fold cross-validation | (1). unbalanced tree=0.88; (2). balanced tree=0.87 |
| Fisher et al, 2015 | CART | a model containing 13 variables (sociodemographic, functional status, clinical variables, comorbidity tier) | / | 0.67 |
| **Study** | **ML based Algorithms** | **Model Description (number of models, number of candidate variables, time)** | **Model Validation** | **Model Discrimination** |
| Jones et al,2019 | GBM | the full model containing 53 variables available at time of discharge (including measures of rehospitalization factors, social situation, cognition, cognition, depression, anxiety, ADLs, pain and other factors); parsimonious model with backward elimination | / | (1). full model = 0.67;  (2). parsimonious model= 0.66 |
| Hogan et al, 2019 | Random Forest | 3 models predicting different timing of readmission outcome containing 131 variables( recipients(demographics, medical, socioeconomic), donors (demographic, medical) and transplant factor(cold ischemia type, number of mismatch), ESRD-related risk factor(dialysis vintage, modality) at time of transplantation | 3-fold cross-validation | (1). 30 days=0.61(0.60, 0.63); (2). 90 days=0.62 (0.61, 0.64); (3). 365 days=0.63 (0.62, 0.64) |
| Zack et al, 2019 | Random forest | a model containing 410 variables (sociodemographic, clinical diagnoses, admission, procedural and in-hospital events) available at discharge | 8-fold cross-validation internally; training/testing split by time: 2004-2008/2009-2013 | 0.90 (0.89-0.91) |
| Mahajan et al, 2016 | RF | two models containing 48 clinical predictors (vitals, lab value, comorbidities) at the time of discharge | / | 0.61 |
| Xue et al, 2019 | Random forest; SVM | three models trained by three different algorithms, and each model include 3 submodels containing different predictors were compared: baseline model (demographic, comorbidity only), FIM model (baseline+ admission FIM), FIM plus model (all 17 predictors). | training/testing split by time: 2011-2015/2016-2017; 10-fold cross-validation | a. SVM: baseline=0.612 (0.564, 0.661), FIM only=0.716 (0.674, 0.758), FIM plus=0.794 (0.752, 0.837); b. RF: baseline=0.625 (0.576, 0.676), FIM only=0.819 (0.767, 0.850), FIM plus=0.813 (0.772, 0.853) |
| **Study** | **ML based Algorithms** | **Model Description (number of models, number of candidate variables, time)** | **Model Validation** | **Model Discrimination** |
| Mahajan et al, 2018 | Boosted trees analysis; Spike-and-slab regression | two models trained by two algorithms containing 56 predictors (from domains of clinical, administrative and psychosocial) at the time discharge and grid search methods were used for parameter tuning for each algorithm | / | a. boosted trees=0.719; b. spike-and-slab regression=0.621 |
| Kalagara et al, 2019 | Supervised gradient boosting machine (GBM) | one model containing all variables (14 patient and operation characteristics, 21 postsurgical complications and 36 comorbidities); one model containing only predischarge variables (including discharge location, comorbidities, ASA classification) | training/testing: 85%/15%; three repeats of a 10-fold cross-validation | (1). Model with all variables=0.8059; (2). Model with only predischarge variables=0.6901 |
| Merrill et al, 2018 | Gradient boosting | one model containing 17 variables (age, elective surgery, sex, insurance, teaching hospital, deficiency anemias, CHF, chronic lung diseases, depression, diabetes, hypertension, obesity, PVD, renal failure and open fracture) following ORIF | training/testing (70%/30%); three repeats of a 10-fold cross-validation | 0.6979 |
| Rojas et al, 2017 | Gradient boosted machine (GBM) | one model containing 117 variables (demographics, vital signs, laboratory values, medications administered during the ICU admission, ICU interventions, nursing documentation, diagnostic tests); a simpler model only containing physiology related variables (age, vital and lab data). | training/testing (60%/40%) internally; external validation | Internal Validation: (1).GBM(72 hours readmission)=0.72 (0.71, 0.75); (2).GBM( ³72 hours readmission)n=0.77 (0.75, 0.78); (3).GBM(Ever readmission)=0.76 (0.75-0.78); External Validation: GBM(Ever readmission)= 0.71 (0.70-0.72) |
| **Study** | **ML based Algorithms** | **Model Description (number of models, number of candidate variables, time)** | **Model Validation** | **Model Discrimination** |
| Chandra et al, 2019 | GBM | one comprehensive model containing 17 variables (patient demographics, medical comorbidity, prior use of healthcare, clinical parameters during the index hospitalization) | out-of-sample 10-fold cross-validation | 0.70 (0.68-0.72) |
| Pakbin et al, 2018 | Gradient descent boosting | 1 model trained by two different algorithms containing 58 variables (admissions, ICU stays, patients, Procedure Events, ICD9 Admission Diagnosis Codes, Chart events, and Lab events. Chart events contains vitals, measurements, and other such time-series events and nursing), and each model included 6 submodels predicting different timing of readmission outcome. | training/testing 80%/20%; stratified k-fold cross-validation | (1).24 hours=0.72 (0.70-0.74); (2). 72 hours =0.76 (0.74-0.77), (3).24-72 hours = 0.75 (0.72-0.78); (4). 7 days=0.77 (0.76-0.78); (5).30 days= 0.75 (0.74-0.77); (6). Bounceback: 0.84 (0.83-0.85) |
| Salem et al, 2018 | Support vector machine | a model containing 14 variables (Scores on the 9 subscales of the BPQ and 5 clinical variables (sex, age, diagnosis, LOS, number of admissions at BPQ baseline) | training/testing split (69.89%/30.11%) | 0.86 |
| Rumshisky et al, 2016 | SVM | 3 models based on different sets of variables(ranging from 4 to 1004): (1)only baseline features (age/ gender/ insurance/Charlson) , (2) baseline features + top-N words (N most informative words from each patient's record), (3)baseline+ topic features derived from the k-topic LDA model | training/testing split (70%/30%); 3-fold cross-validation | (1). baseline clinical model= 0.618; (2). baseline features+ top-N bag-of-words features words= 0.682; (3). baseline+ topic features derived from the k-topic LDA model= 0.784 |
| **Study** | **ML based Algorithms** | **Model Description (number of models, number of candidate variables, time)** | **Model Validation** | **Model Discrimination** |
| Turgeman et al, 2016 | DT; SVM; DT ensembled with SVM; other ensembled algorithms | Multiple standalone models based on different learning algorithms and other mixed-ensemble models (combining C5.0 tree with SVM) containing 38 variables (including demographic and historical variables, lab values, comorbidities) | training/testing (75%/25%); 10-fold cross-validation | AUC: Stand-alone models: a. SVM=0.643; b. Naïve-Bayes net=0.676; c. Neural Net=0.639; Ensemble models: e. DT C5+SVM=0.693; f. Naive Net+ Neural Net=0.65; g. Naive net+ SVM=0.649; h. Neural-Net+ SVM=0.577  Precision: Stand-alone models:  a. SVM= 0.286; b. Naïve-Bayes net= 0.250; c. Neural Net=0.053; Ensemble models: e. DT C5+SVM= 0.197; f. Naive Net+ Neural Net=0.234; g. Naive net+ SVM=0.263; h. Neural-Net+ SVM=0.287 |
| Povalej et al, 2017 | Regularized Logistic Regression, RF, XGBoost | 3 models trained by 3 algorithms; each model included 5 submodels based on five datasets of different sample size stratified by the number of available hospitalizations per patient of datasets, and containing 29.50 to 184.960 variables (demographic, information about the hospital stay, primary diagnosis, and procedures during hospitalization) | 100 repeated 10-fold cross-validation | a. LASSO(D1)=0.639 (0.637, 0.642); LASSO(D2)=0.673 (0.67, 0.675); LASSO(D3)= 0.672 (0.669, 0.675); LASSO(D4)=0.668 (0.664, 0.671); LASSO(D5)=0.678 (0.673, 0.683); b. RF(D1)=0.655 (0.653, 0.658); RF(D2)=0.678 (0.675, 0.68); RF(D3)=0.686 (0.683, 0.69); RF(D4)=0.694 (0.691, 0.698); RF(D5)=0.696 (0.691, 0.701); c. XGBoost (D1)=0.645 (0.642, 0.647); XGBoost (D2)=0.673 (0.67, 0.675); XGBoost (D3)=0.675 (0.672, 0.679); XGBoost (D4)=0.686 (0.682, 0.689); XGBoost (D5)=0.686 (0.681, 0.691) |
| Mahajan et al, 2017 | Regularized Logistic Regression | One full model containing 59 predictor variables(clinical, administrative (comorbidities and demographic variables), and psychosocial domain (psychiatric comorbidities including substance abuse and other variables indicating patient’s behavioral and social situation)), and another 4 submodels with sub-datasets with only psychosocial, clinical, administrative or combined clinical and administrative domain variables, respectively. | 10-fold cross-validation | (1). Psychosocial only Model=0.50 (0.49, 0.51); (2).clinical only model=0.65 (0.64, 0.66); (3).administrative only model=0.71 (0.69, 0.72); (4).combined clinical and administrative model= 0.77 (0.75, 0.78); (5).full model=0.84 (0.83, 0.85) |
| **Study** | **ML based Algorithms** | **Model Description (number of models, number of candidate variables, time)** | **Model Validation** | **Model Discrimination** |
| Nakamura et al, 2019 | L2-regularized logistic regression; CNN | Three models with first two models using L2-regularized logistic regression, with a core model containing 121 variables (including age, gender, chronic conditions and primary diagnosis were developed using two algorithms, and an improved multivariable model containing 121 variables with addition of initial vital signs and lab variables; and a third model using CNN. | / | (1). core mode = 0.713 (0.706, 0.719); (2). multivariable model (with addition of clinical variables) = 0.722 (0.715, 0.728); (3). AUC for CNN model not reported |
| Ehwerhemuepha et al, 2020 | Least absolute shrinkage and selection operator regression model | LASSO was used to train the model at the time discharge based on 86 variables from such as demographics, SDoH, health care use, diagnoses, medications, severity of illness, acuity, psychosocial variables, discharge related variables (season of discharge, day of discharge week), and primary care related (primary care no show, primary care visit cancellations). | training/testing split (50%/50%) | 0.778 (0.763–0.793) |
| Tong et al, 2016 | Least absolute shrinkage and selection operator logistic regression; AdaBoost | two models using LASSO and AdaBoost algorithms; and each model contains four submodels, applied in samples with various sizes. | training/testing split (50%/50%) | LASSO: size~2500=0.703 (0.680, 0.727), size~5,000= 0.703 (0.690, 0.717), size~20,000= 0.735 (0.728, 0.743), size~80,000=0.737 (0.733, 0.741); b. AdaBoost: size~2500=0.676 (0.650, 0.703), size~5,000= 0.694 (0.678, 0.709), size~20,000= 0.735 (0.728, 0.743), size~80,000=0.737 (0.733, 0.741) |
| **Study** | **ML based Algorithms** | **Model Description (number of models, number of candidate variables, time)** | **Model Validation** | **Model Discrimination** |
| McKinley et al, 2018 | K nearest neighbor; randomized K-nearest neighbor (rKNN), support vector machine (SVM), random forest,  gradient boosting machine (GBM), and Lasso regression | 6 different ML algorithms-based models containing 29 variables (demographic, social and clinical) were trained on the propensity score matched data combining the control and intervention groups. | training/test split (66.7%/33.3%); 10-fold cross-validation | a. KNN (combined group) = 0.768, KNN (balanced group) = 0.70; b. rKNN (combined group) = 0.469, c. SVM=0.496; d. random forest=0.616; e. GBM=0.589; f. LASSO=0.576 |
| Kulkarni et al, 2016 | NN, DT | 2 different ML algorithm based models applying data of all patient cohorts, and each containing admission Model(AM)and discharge model (DM), with AM containing 6 variables, DM containing 8 variables. | training/validation split (75%/25%) | a. NN(AM)=0.78, NN(DM)=0.79; b. DT(AM)=0.72, DT(DM)=0.77 |
| Goyal et al, 2019 | ANNs, RF, gradient boosting machines (GBMs), naïve Bayes, penalized linear discriminant analysis, elastic-net GLM | 6 different ML algorithm based models containing 27 variables (patient characteristics (age, race, sex, BMI), behavioral (smoking), medical history (diabetes, HTN, COPD, bleeding disorder, dyspnea), prior medication use (chronic corticosteroid use)) using fter discharge using retrospective analysis. | 10-fold cross-validation | a. ANN=0.63; b. RF=0.64; c. GBM=0.66; d. VarBayes=0.66; e.pLDA=0.66; f. GLMnet=0.66 |
| Garcia-Acre et al, 2018 | NNs; RF; GBM; SVM | 4 different ML algorithm containing 17 variables(age, LoS, admission type, behavioral health comorbidity index, marital status, discharge disposition, payer, race, sex, disease severity index and etc..) applied to 5 different patient cohorts (with AMI, pneumonia, COPD, CHF, type 2 diabetes) after discharge using retrospective analysis. | training/testing split (66.7%/33.3%); 10-fold cross-validation | a. NN(AMI=0.7518, NN(pneumonia)=0.6546, NN(COPD)=0.6988, NN(CHF)=0.6248,NN(diabetes)=0.6957; b. RF(AMI)=0.747,RF(pneumonia)=0.6325, RF(COPD)=0.6886, RF(CHF)=0.6,RF(diabetes)=0.6699829; c. GBM(AMI)=0.7510, GBM(pneumonia)=0.6442, GBM(COPD)=0.6850, GBM(CHF)=0.6314,GBM(diabetes)=0.6829; d. SVM(AMI)=0.7074, SVM(pneumonia)=0.5598, SVM(COPD)=0.611, SVM(CHF)=0.5728,SVM(diabetes)=0.6123; |
| **Study** | **ML based Algorithms** | **Model Description (number of models, number of candidate variables, time)** | **Model Validation** | **Model Discrimination** |
| Mortazavi et al, 2016 | Random forests (RF), Boosting, RF combined hierarchically with Support Vector Machines (SVM), | 3 different ML algorithms-based models containing 236 variables (medical record abstractions, hospital laboratory results, physical examination information as well as quality of life, socioeconomic, and demographic information) after discharge using retrospective analysis and each containing 3 submodels with different prediction outcomes. | 100 repeated random training/test split (50%/50%) | a. RF(30 day readmission)0.628 (0.624, 0.633), RF(180 day readmission)=0.654 (0.650, 0.657) , RF(HF only readmission)=0.669 (0.661, 0.676); b. Boosting(30 day readmission)= 0.613 (0.607, 0.618), boosting (180 day readmission)=, boosting (HF only readmission)=0.678 (0.670, 0.687). c. RF combined SVM (30 day readmission) = 0.583 (0.579, 0.587), RF combined SVM (180 day readmission) =0.654 (0.650, 0.657), RF combined SVM(HF only readmission)=0.657 (0.652, 0.661) |
| Frizzell et al, 2017 | Tree augmented naïve Bayesian network, RF, GBM, LASSO | 4 different ML algorithms-based models containing 93 candidate variables (demographics, SES, medical history, characterization of HF, admission and discharge medications, vitals, weights, selected lab and discharge interventions) after discharge | training/test split (70%/30%) | a. tree-augmented naïve Bayesian network=0.618; b. RF=0.607; c. GBM=0.614; d. LASSO logit= 0.618 |
| Eckert et al, 2018 | DT; AdaBoost; RF | 3 different ML algorithms based models containing 54 variables (patient comorbidities, health care utilization elements, and pharmaceutical details, vital signs, and lab values) applying on the retrospective cohort after discharge; and another 3 revised models containing additional features (CCI, military rank, prior and current medication use) applying pediatric patients (aged <18) and among adults (aged >=18) | retrospective training (01/2014-01/2016)/prospective testing (06/2017-08/2017)/retrospective testing (01/2014-06/2017); 10-fold cross-validation | AUC: a. AdaBoost (retrospective analyses) =0.68, AdaBoost (prospective validation) = 0.64, AdaBoost (retrospective revised model) = 0.76; b. DT (retrospective analyses) = 0.60; c. RF (retrospective analyses) = 0.65;  Precision:  a. AdaBoost (retrospective analyses) = 0.18, AdaBoost (prospective validation) = 0.15, AdaBoost (retrospective revised model) = 0.23; b. DT (retrospective analyses) = 0.21; c. RF (retrospective analyses) = 0.20;  Recall: a. AdaBoost (retrospective analyses) = 0.73, AdaBoost (prospective validation) = 0.66, AdaBoost (retrospective revised model) = 0.76; b. DT (retrospective analyses) = 0.65; c. RF (retrospective analyses) =0.71; |
| **Study** | **ML based Algorithms** | **Model Description (number of models, number of candidate variables, time)** | **Model Validation** | **Model Discrimination** |
| Lodhi et al, 2017 | DT; Naïve-Bayes; KNN; SVM | 4 different ML algorithms-based models containing x variables (patient, nurse staff variables, diagnoses, interventions) at the time of discharge. | 10-fold cross-validation | AUC: a. DT=0.78; b. NB=0.71; c. KNN(K=2) =0.62, KNN(K=5) = 0.69, KNN(K=10):0.67; d. SVM=0.65;  Precision:  a. DT=0.725; b. NB=0.674; c. KNN(K=2) =0.608, KNN(K=5) = 0.605, KNN(K=10)=0.591; d. SVM=0.591;  Recall:  a. DT=0.774; b. NB=0.727; c. KNN(K=2) =0.841, KNN(K=5) = 0.851, KNN(K=10)=0.823; d. SVM=0.807;  F-1 Score:  a. DT=0.75; b. NB=0.70; c. KNN(K=2) =0.71, KNN(K=5) = 0.71, KNN(K=10)=0.69; d. SVM=0.68; |
| Jamei et al, 2017 | ANN, Random forests, and Neural networks | several different ML based models containing 1667 features using real-time EHR during inpatient stays or at time of discharge and then the model was retained with only top N features retained; and then NN models and LACE models were built across specific age group, hospitals and medical conditions | 5-fold cross-validation | AUC:  a. 2-layer NN (1667 features) =0.78, 2 layer NN (500 features) =0.77, 2 layer NN (features)=0.76; b. RF=0.77;  Precision:  a. 2-layer NN (1667 features) =0.24, 2 layer NN (500 features) =0.22, 2 layer NN (100 features)=0.22; b. RF=0.23;  Recall:  a. 2-layer NN (1667 features) =0.60, 2 layer NN (500 features) =0.61, 2 layer NN (features)=0.58; b. RF=0.57; |
| Welchowski et al, 2016 | Kernel deep stacking networks; RF, H20 deep learning, darch algorithms | KDSN model was trained on 31 predictors of 4 domains (demographic, clinical variables, medical features, hospital characteristic, readmission information) | 10-fold cross-validation | a. KDSN= 0.61 (0.59, 0.63); b.RF=0.53 (0.52, 0.55); c.darch=0.59(0.58, 0.61); d.H2O= 0.57 (0.56, 0.59). |
| **Study** | **ML based Algorithms** | **Model Description (number of models, number of candidate variables, time)** | **Model Validation** | **Model Discrimination** |
| Wang et al. 2018 | Convolutional neural networks; Support Vector Machine (SVM), DT, RF, ANN | CNN with cost sensitive (CSDNN) was developed separately based on 34 variables (demographics, vital signs, lab values) from GHW s data, and 76 variables (pre-operation and intra-operation vital signs) from ORP data. | training/validation/test split: 60%/15%/25% | AUC: a. CSDNN(30-d readmission on GHWs)=0.70, CSDNN(60-d readmission on GHWs)=0.71, CSDNN(1 year readmission on ORP)= 0.76, CSDNN(30-d readmission on ORP)=0.73; b. SVM(30-d readmission on GHWs)=0.53, SVM(60-d readmission on GHWs)=0.60; SVM(1 year readmission on ORP)= 0.58, SVM(30-d readmission on ORP)=0.65; c. DT (30-d readmission on GHWs)=0.61, DT(60-d readmission on GHWs)=0.59; DT(1 year readmission on ORP)= 0.63, DT(30-d readmission on ORP)=0.63; d.RF (30-d readmission on GHWs)=0.57, RF(60-d readmission on GHWs)=0.61; RF(1 year readmission on ORP)= 0.55, RF(30-d readmission on ORP)=0.64; e.ANN(30-d readmission on GHWs)=0.62, ANN(60-d readmission on GHWs)=0.66; ANN(1 year readmission on ORP)= 0.71, ANN(30-d readmission on ORP)=0.69;  F1-Score:  a. CSDNN(30-d readmission on GHWs)=0.44, CSDNN(60-d readmission on GHWs)=0.45, CSDNN(1 year readmission on ORP)= 0.54, CSDNN(30-d readmission on ORP)=0.64; b. SVM(30-d readmission on GHWs)=0.15, SVM(60-d readmission on GHWs)=0.31; SVM(1 year readmission on ORP)= 0.26, SVM(30-d readmission on ORP)=0.00; c. DT (30-d readmission on GHWs)=0.00, DT(60-d readmission on GHWs)=0.00; DT(1 year readmission on ORP)= 0.44, DT(30-d readmission on ORP)=0.37; d.RF (30-d readmission on GHWs)=0.19, RF(60-d readmission on GHWs)=0.37; RF(1 year readmission on ORP)= 0.51, RF(30-d readmission on ORP)=0.38; e.ANN(30-d readmission on GHWs)=0.36, ANN(60-d readmission on GHWs)=0.37; ANN(1 year readmission on ORP)= 0.48, ANN(30-d readmission on ORP)=0.55; |
| **Study** | **ML based Algorithms** | **Model Description (number of models, number of candidate variables, time)** | **Model Validation** | **Model Discrimination** |
| Hopkins et al, 2019 | Deep neural network (DNN) classification | Two DNN models was trained separately on 177 input variables (including demographics, clinical characteristics, comorbidities, preoperative lab values, operative procedures and postoperative complications) and on 103 variables with (all the above variables excepting operative and postoperative variables) at the time of discharge. | training/test split: 75%/25% | (1). DNN (all inputs) =0.812, (2). DNN (preoperative inputs only) =0.58 |
| Golas et al, 2018 | Deep unified network; GBM | deep unified networks with 6 different deep learning architectures (DNN, deep autoencoder, ) were trained on 3512 variables from over 25 feature categories (including demographic, socioeconomic, admission related data, diagnosis, lab data, medication data, procedure data, unstructured patient notes) were used for model derivation at time of discharge. | 10-fold cross-validation | AUC:  a. Deep Unified Network= 0.705 (0.69, 0.72); b. Maxout networks=0.695 (0.679, 0.711); c. Gradient boosting=0.650(0.639, 0.611);  Precision:  a. Deep Unified Network= 0.360 ± 0.015; b. Maxout networks= 0.354 ± 0.016; Gradient boosting= 0.325 ± 0.008;  Recall:  a. Deep Unified Network= 0.652 ± 0.036; b. Maxout networks= 0.631 ± 0.016; c. Gradient boosting= 0.615 ± 0.032;  F-1 Score: a. Deep Unified Network= 0.464 ± 0.013; b. Maxout networks= 0.454 ± 0.016; c. Gradient boosting= 0.425 ± 0.010; |
| Reddy et al, 2018 | Recurrent neural network; regularized regression, artificial neural networks | Three RNN models (RNN, RNN-LTSM, RNN-GRU) were trained on the longitudinal data of each time point for each patients of 20 variables from 6 categories (demographics, clinical diagnosis, healthcare setting, insurance, disease status, hospital stay derived) | training/testing split: 70%/30% | a. Long Short-Term Memory (LSTM)=0.7; b. Gated Recurrent Unit (GRU)=0.70; c. Simple Recurrent Neural Network (RNN)=0.69; d. Regularized/Penalized Logistic Regression=0.63; e. Artificial Neural Network (ANN)=0.66 |
| **Study** | **ML based Algorithms** | **Model Description (number of models, number of candidate variables, time)** | **Model Validation** | **Model Discrimination** |
| Xiao et al, 2018 | Hybrid Topic Recurrent Neural Network (TopicRNN) mode; RNN (GRU), RETAIN, GRU+Word2Vec | a CONTENT model ( a hybrid DL model combining topic modeling and RNN) with word embedding via word2vec and size of 200 hidden layers of RNN was proposed to capture multiple input variables (global context (the general condition, chronic disease, comorbidities), and local context(the short disease progressions)); and word embedding sequences were used as inputs for clinical variables (e.g. diagnoses, procedures, medications, etc.) and the target variable (i.e., readmission) | training/validation/test split: 74.17%/12.98%/12.85% | a. Content Model (real EHR)=0.61 03 (0.5973 0.6233), Content Model(synthetic EHR)= 0.6886 (0.6812 ,0.696); b. GRU (real EHR)=0.5998 (0.5874, 0.6122), GRU (synthetic HER)=0.6881 (0.6833, 0.6929); c. GRU+Word2Vec (real EHR)=0.5616 (0.5459, 0.5773), GRU+Word2Vec (synthetic HER)=0.6836 (0.6805, 0.6867); d. RETAIN Model (real EHR)=0.5707 (0.5567, 0.5847), RETAIN Model(synthetic HER)=0.6927 (0.6926, 0.6928) |
| Lin et al, 2019 | Recurrent neural network (RNN) architecture with long short-term memory (LSTM); LASSO, NB, RF, SVM | 4 RNN_LSTM models were sequentially built upon addition of chart events(CE), ICD-9 and demographics(D); 3 RNN related models ( RNN alone, two bidirectional RNN combination with CNN) were trained to incorporate 331 variables of 3 feature categories (demographic, chart events, ICD-9 embeddings) | training/validation/test split: 80%/10%/10%); 5- fold cross validation | AUC:  a. LSTM+CNN(L48-h CE+ICD-9+D)=0.791 (0.784, 0.798 ); b. LSTM(L48-h CE+ICD-9+D)=0.787 (0.771–0.802); c. RF =0.714(0.703, 0.725); d. NB= 0.709 (0.702–0.716);e. LASSO=0.777 (0.765–0.789); f. SVM= 0.779 (0.768–0.789);  Recall:  a. LSTM+CNN(L48-h CE+ICD-9+D)= 0.710  (0.648–0.771); b. LSTM(L48-h CE+ICD-9+D)= 0.733  (0.698–0.768); c. RF = 0.565  (0.550–0.580); d. NB= 0.709 0.509 (0.479–0.540);e. LASSO= 0.680 (0.662–0.697); f. SVM= 0.509 (0.479–0.540); |
| **Study** | **ML based Algorithms** | **Model Description (number of models, number of candidate variables, time)** | **Model Validation** | **Model Discrimination** |
| Allam et al, 2019 | CNN, RNN, RNNSS, Neutral CRF, CRF Only, RNNCRF; MLP, LASSO | Multiple NN models (RNN, CNN) were implemented with temporal information in the patient's trajectories and other 150 input features (including sociodemographic, hospitalization events, top diagnosis and procedures and the payment source) | stratified 5-fold cross-validation | a.CNN=0.619 (0.616, 0.622); b. CNN-Wide=0.632 (0.629, 0.635); c.RNN=0.636 (0.633, 0.638); d.RNNSS=0.625 (0.623 0.628); e.Neural CRF=0.634 (0.631, 0.637); f. CRF Only=0.630 (0.627, 0.633); g.RNNCRF=0.642(0.640, 0.645); h. MLP= 0.628 (0.625, 0.631); i.LASSO=0.643 (0.640, 0.646) |
| Min et al. 2019 | CNN, RNN, LSTM, GRU; RF, SVM, GBDT, MLP | Deep learning models were constructed to incorporate contextual event embedding, time fusion, and attention mechanisms based on 38 variables of 5 domains (demographics, medication, service location, diagnosis and procedure); other ML algorithms were used to incorporate knowledge drive features(including 4 features(number of procedures during hospital stays, number of hospital admissions during previous year, the number of hospital stays with >=5 days, index admission type) from Hospital score, 4 features (LoS, Acute admission, CCI, number of ED within 6 months) from LACE, other 12 important features((age, gender, LoS, number of admissions in previous year, total length of all stays in previous year, number of all kinds of admissions)), or data-driven features(diagnosis, procedures, pharmacy, locations, Bag-of-Words, Boolean Bag-of-Words, term frequency-inverse document frequency)). | 5-fold cross validation | a. RF (one year)=0.636, RF(Full history)=0.624; b. SVM (one year)=0.612, SVM (Full history)=0.643, c. GBDT (one year)=0.653, GBDT (Full history)=0.654; d. MLP (one year)=0.571, MLP (Full history)=0.627; e. LR-l1 regularization(one year)=0.616, LR-l1 regularization(Full history)=0.644; f. LR-l2 regularization (one year)=0.617, LR-l2 regularization(Full history)=0.645 |
| **Study** | **ML based Algorithms** | **Model Description (number of models, number of candidate variables, time)** | **Model Validation** | **Model Discrimination** |
| Rajkomar et al, 2018 | Ensemble of RNN (Weighted Recurrent neural network model), Feedforward Model with Time-Aware Attention (TANNs) and NN with boosted embedded time-series model. | Three deep learning neural network model architectures were trained based on tens of thousands of predictors (Patient, Encounter, Medication, Observation (e.g. vital signs and nursing documentation), Composition (e.g. notes),Conditions (i.e. diagnoses), Medication Administration, Medication Order, Procedure Request, and Procedure) at multiple time points (including before admission, at admission, 24 h after admission and at discharge), and the results of each architecture were combined using ensemble learning. | training/validation/test split: 80%/10%/10% | (1). Hospital A: at admission=0.73(0.71-0.74); at 24 h after admission=0.74(0.72-0.75); at discharge=0.77(0.75-0.78); (2). Hospital B: at admission=0.72(0.71-0.73); at 24 h after admission=0.73(0.72-0.74); at discharge=0.76(0.75-0.77) |
| Abbreviations: ML: machine learning; NNs: neural networks; RF: random forest, DT: decision tree; SVM: support vector machine; CART: Classification And Regression Trees; GBM: gradient boosting machines; GDB: gradient descent boosting; MLPs: Multilayer Perceptron; CNNs: Convolutional Neural Networks; RNNs: Recurrent Neural Networks; LSTM: Long short-term memory; GRU: Gated recurrent unit; CRF: Conditional random field; >: means better performances | | | | |

**Table S3. Summary of Variables Included in ML Models (N=43 studies)**

| **Study** | **Demogr-aphic** | **Social Determinants** | **Primary Diagnosis or comorbidity index** | **Illness Severity** | **Mental Health Comorbidities** | **Overall Health Status and Functional Status** | **Pre-Index Utilization** |
| --- | --- | --- | --- | --- | --- | --- | --- |
| **Domains** | **Age, gender, race, living area** | **Income/Insurance/Employment, education, marital, caregiver, access to care, discharge location** | **Specific medical diagnoses or comorbidity index** | **Severity index, lab findings, others** | **Mental Illness,**  **Substance/Alcohol Use** | **ADL dependent, and mobility, Self-reported Health, QoL, Cognitive impairment,**  **Visual, or hearing impairment** | **ER Visits, Hospitalizations, Length of Stay, Clinical Visits/Missed Clinic Visits** |
| Kang et al, 2016 | y | y | Y | n | n | y | n |
| Brom et al, 2019 | y | y | Y | n | n | n | y |
| Yeo et al, 2016 | y | n | Y | n | n | y | y |
| Edgcomb et al, 2019 | y | y | Y | n | y | n | y |
| Fisher et al, 2015 | y | y | Y | n | n | y | y |
| Jones et al,2019 | y | n | Y | n | y | y | y |
| Hogan et al, 2019 | y | y | Y | n | y | n | y |
| Zack et al, 2019 | y | y | Y | n | n | n | y |
| Mahajan et al, 2016 | y | n | Y | n | n | n | n |
| Xue et al, 2019 | y | y | Y | n | n | y | y |
| Mahajan et al, 2018 | y | y | Y | y | y | n | y |
| Kalagara et al, 2019 | y | y | Y | y | n | y | y |
| Merrill et al, 2018 | y | y | Y | n | n | n | y |
| Rojas et al, 2017 | y | n | Y | y | y | n | y |
| Chandra et al, 2019 | y | y | Y | y | n | n | y |
| Pakbin et al, 2018 | y | n | Y | y | n | n | y |
| Salem et al, 2018 | y | n | Y | y | y | n | y |
| Rumshisky et al, 2016 | y | y | Y | n | y | n | y |
| Turgeman et al, 2016 | y | n | Y | y | n | n | y |
| Povalej et al, 2017 | y | n | Y | n | n | n | y |
| Mahajan et al, 2017 | y | y | Y | y | y | n | y |
| **Study** | **Demogr-aphic** | **Social Determinants** | **Primary Diagnosis or comorbidity index** | **Illness Severity** | **Mental Health Comorbidities** | **Overall Health Status and Functional Status** | **Pre-Index Utilization** |
| **Domains** | **Age, gender, race, living area** | **Income/Insurance/Employment, education, marital, caregiver, access to care, discharge location** | **Specific medical diagnoses or comorbidity index** | **Severity index, lab findings, others** | **Mental Illness,**  **Substance/Alcohol Use** | **ADL dependent, and mobility, Self-reported Health, QoL, Cognitive impairment,**  **Visual, or hearing impairment** | **ER Visits, Hospitalizations, Length of Stay, Clinical Visits/Missed Clinic Visits** |
| Nakamura et al, 2019 | y | n | Y | y | n | n | y |
| Ehwerhemuepha et al, 2020 | y | y | Y | y | y | n | y |
| Tong et al, 2016 | y | y | Y | y | y | y | y |
| McKinley et al, 2018 | y | n | Y | n | n | y | y |
| Kulkarni et al, 2016 | y | y | Y | n | n | n | y |
| Goyal et al, 2019 | y | y | Y | y | n | y | n |
| Garcia-Acre et al, 2018 | y | y | Y | y | n | n | y |
| Mortazavi et al, 2016 | y | y | Y | n | n | y | y |
| Frizzell et al, 2017 | y | y | Y | y | n | n | y |
| Eckert et al, 2018 | y | y | Y | y | n | n | y |
| Lodhi et al, 2017 | y | n | Y | n | y | n | y |
| Jamei et al, 2017 | y | y | Y | y | n | n | y |
| Welchowski et al, 2016 | y | n | Y | n | n | n | y |
| Wang et al. 2018 | y | n | Y | y | n | n | y |
| Hopkins et al, 2019 | y | n | Y | y | n | n | y |
| Golas et al, 2018 | y | y | Y | y | y | n | y |
| Reddy et al, 2018 | y | y | Y | n | n | n | y |
| Xiao et al, 2018 | y | n | Y | y | n | n | y |
| Lin et al, 2019 | y | y | Y | y | n | n | y |
| Allam et al, 2019 | y | y | Y | n | n | n | y |
| Min et al. 2019 | y | n | Y | y | n | n | y |
| **Study** | **Demogr-aphic** | **Social Determinants** | **Primary Diagnosis or comorbidity index** | **Illness Severity** | **Mental Health Comorbidities** | **Overall Health Status and Functional Status** | **Pre-Index Utilization** |
| **Domains** | **Age, gender, race, living area** | **Income/Insurance/Employment, education, marital, caregiver, access to care, discharge location** | **Specific medical diagnoses or comorbidity index** | **Severity index, lab findings, others** | **Mental Illness,**  **Substance/Alcohol Use** | **ADL dependent, and mobility, Self-reported Health, QoL, Cognitive impairment,**  **Visual, or hearing impairment** | **ER Visits, Hospitalizations, Length of Stay, Clinical Visits/Missed Clinic Visits** |
| Rajkomar et al, 2018 | y | n | Y | y | n | n | y |
| Abbreviations: y=Yes; n=No | | | | | | | |

**Table S4. Summary of Other Performances Measures of ML Models (N=43 studies)**

| **Study** | **Accuracy** | **Sensitivity** | **Specificity** | **Calibration** | **Methods to address imbalance data** | **Other Measures: Precision, Recall, or F1 Score** |
| --- | --- | --- | --- | --- | --- | --- |
| Kang et al, 2016 | n | y | y | n | n | N |
| Brom et al, 2019 | n | n | n | n | n | N |
| Yeo et al, 2016 | n | y | y | n | n | N |
| Edgcomb et al, 2019 | y | y | y | n | n | N |
| Fisher et al, 2015 | n | n | n | y | n | N |
| Jones et al,2019 | n | n | n | y | n | N |
| Hogan et al, 2019 | y | n | n | n | n | N |
| Zack et al, 2019 | n | y | y | n | n | N |
| Mahajan et al, 2016 | y | y | y | n | n | N |
| Xue et al, 2019 | n | n | n | n | n | N |
| Mahajan et al, 2018 | n | n | n | n | n | N |
| Kalagara et al, 2019 | y | y | y | n | Y: SMOTE | N |
| Merrill et al, 2018 | y | y | y | n | n | N |
| Rojas et al, 2017 | n | y | y | n | n | N |
| Chandra et al, 2019 | n | y | y | n | n | N |
| Pakbin et al, 2018 | n | n | n | y | n | N |
| Salem et al, 2018 | y | y | y | n | n | N |
| Rumshisky et al, 2016 | y | y | y | n | n | N |
| Turgeman et al, 2016 | y | y | y | n | Y: Modification to the Classifers Using New Optimization Methods for Boosting | Y: Only Precision |
| Povalej et al, 2017 | n | y | y | n | n | N |
| Mahajan et al, 2017 | n | n | n | n | n | N |
| Nakamura et al, 2019 | n | n | n | n | n | N |
| Ehwerhemuepha et al, 2020 | y | y | y | n | n | N |
| Tong et al, 2016 | n | n | n | n | n | N |
| McKinley et al, 2018 | y | n | n | n | n | N |
| Kulkarni et al, 2016 | y | n | n | n | n | N |
| Goyal et al, 2019 | y | y | y | n | n | N |
| Garcia-Acre et al, 2018 | n | n | n | n | Y: SMOTE | N |
| Mortazavi et al, 2016 | n | y | y | n | n | N |
| Frizzell et al, 2017 | y | n | n | y | n | N |
| Eckert et al, 2018 | y | y | y | n | n | Y: Precision and Recall |
| **Study** | **Accuracy** | **Sensitivity** | **Specificity** | **Calibration** | **Methods to address imbalance data** | **Other Measures: Precision, Recall, or F1 Score** |
| Lodhi et al, 2017 | y | n | n | n | n | Y: Precision and Recall |
| Jamei et al, 2017 | n | y | n | n | n | Y: Precision and Recall |
| Welchowski et al, 2016 | n | n | n | n | n | N |
| Wang et al. 2018 | y | y | y | n | Y:Cost-sensitive prediction formulation | Y: F1 Score |
| Hopkins et al, 2019 | y | y | y | n | n | N |
| Golas et al, 2018 | n | n | n | n | n | Y: Precision, Recall and F1 Score |
| Reddy et al, 2018 | y | y | y | n | Y: SMOTE | N |
| Xiao et al, 2018 | n | n | n | n | n | N |
| Lin et al, 2019 | y | y | y | n | n | Y: Only Recall |
| Allam et al, 2019 | n | n | n | n | Y: Inverse weighting to training datasets | N |
| Min et al. 2019 | n | n | n | n | n | N |
| Rajkomar et al, 2018 | n | n | n | n | n | N |
| Abbreviations: n=No; y=Yes | | | | | | |

**Table S5. Risk of Bias Assessment (N=43 studies)**

| **Study** | **Study Population*** | **Study Attrition†** | **Prognostic Measure‡** | **Outcome Measurement§** | **Study Confoundingǂ** | **Statistical Analysis and Reporting•** | **Quality**  **Assessment** |
| --- | --- | --- | --- | --- | --- | --- | --- |
| **Orignal Quips** | **The study sample adequately represents the population of interest** | **The study data available(i.e, participants not lost to follow up) adequately represent the study sample** | **The PF is measured in a similar way for all participants** | **The outcome of interest is measured in a similar way for all participants** | **Important potential confounding factors are appropriately accounted for** | **The statistical analysis is appropriate, and all primary outcomes are reported** |  |
| **Tailor to our study** | **Is there an adequate description of study population** | **Did the study provide an adequate description of follow-up information, e.g. describing about any method for handling loss-to-follow-up or deaths?** | **Did the study provide an adequate description of measurement of prognostic factors, e.g. describing about any imputation method for handling missing data?** | **Is there a clear definition of the readmission outcome?** | **Did the study accounted for potential confounding factors from more than three of following domains, such as demographic factors, social determinants of health (SDoH), primary diagnosis or comorbidity index, illness severity, mental health comorbidities, overall health and functional status, prior use of medical services hospitalizations?** | **Did the study conduct any model validation procedure?** |  |
| Kang et al, 2016 | yes | partly-did not mention about handling of loss to follow due to death. | yes | yes | Parly, did not include pre-index utilization (such as length of stay) | yes | Moderate |
| Brom et al, 2019 | yes | partly-did not mention about handling of loss to follow due to death. | yes | yes | yes | yes | High |
| Yeo et al, 2016 | yes | partly-did not mention about handling of loss to follow due to death. | yes | yes | yes | no, did not provide any validation methods | Moderate |
| **Study** | **Study Population*** | **Study Attrition†** | **Prognostic Measure‡** | **Outcome Measurement§** | **Study Confoundingǂ** | **Statistical Analysis and Reporting•** | **Quality**  **Assessment** |
| Edgcomb et al, 2019 | yes | partly-did not mention about handling of loss to follow due to death. | yes | yes | yes | yes | High |
| Fisher et al, 2015 | yes | yes | yes | yes | yes | no, did not provide any validation methods | High |
| Jones et al,2019 | yes | yes | yes | yes | yes | no, did not provide any validation methods | High |
| Hogan et al, 2019 | yes | yes | yes | yes | yes | yes | High |
| Zack et al, 2019 | yes | yes | yes | yes | yes | yes | High |
| Mahajan et al, 2016 | Partly, did not describe the study period | Partly-did not mention about handling of loss to follow due to death. | yes | yes | Parly, did not include pre-index utilization (such as length of stay) | no, did not provide any validation methods | Low |
| Xue et al, 2019 | yes | partly-did not mention about handling of loss to follow due to death. | yes | yes | yes | yes | High |
| Mahajan et al, 2018 | yes | partly-did not mention about handling of loss to follow due to death. | yes | yes | Yes | yes | High |
| Kalagara et al, 2019 | yes | partly-did not mention about handling of loss to follow due to death. | yes | yes | yes | yes | High |
| **Study** | **Study Population*** | **Study Attrition†** | **Prognostic Measure‡** | **Outcome Measurement§** | **Study Confoundingǂ** | **Statistical Analysis and Reporting•** | **Quality**  **Assessment** |
| Merrill et al, 2018 | yes | yes | parly, though it provided a description of PF, but it did not provide methods of PF measurement, and it did not provide handling continuous variables, and did not mention about imputations | yes | yes | yes | High |
| Rojas et al, 2017 | yes | yes | yes | yes | Yes | yes | High |
| Chandra et al, 2019 | yes | yes | yes | yes | Yes | yes | High |
| Pakbin et al, 2018 | yes | partly-did not mention about handling of loss to follow due to death. | yes | yes | yes | yes | High |
| Salem et al, 2018 | yes | Partly-did not mention about handling of loss to follow due to death. | yes | partly-though it mentioned about 30-day readmission, however, it did not contain description of definition of readmission outcome | Yes | yes | Low |
| Rumshisky et al, 2016 | yes | yes | yes | partly-though it mentioned about 30-day readmission, however, it did not contain description of definition of readmission outcome | Yes | yes | High |
| Turgeman et al, 2016 | yes | yes | yes | no, did not provide timing of readmission outcome and did not provide the definition of readmission outcome | Yes | yes | High |
| **Study** | **Study Population*** | **Study Attrition†** | **Prognostic Measure‡** | **Outcome Measurement§** | **Study Confoundingǂ** | **Statistical Analysis and Reporting•** | **Quality**  **Assessment** |
| Povalej et al, 2017 | yes | yes | yes | yes | Yes | yes | High |
| Mahajan et al, 2017 | yes | yes | yes | yes | Yes | yes | High |
| Nakamura et al, 2019 | yes | yes | yes | yes | Yes | no, did not provide any validation methods | High |
| Ehwerhemuepha et al, 2020 | yes | yes | yes | yes | Yes | yes | High |
| Tong et al, 2016 | yes | yes | yes | yes | Yes | yes | High |
| McKinley et al, 2018 | yes | partly-did not mention about handling of loss to follow due to death. | yes | yes | Yes | yes | High |
| Kulkarni et al, 2016 | partly, described patients admitted to private health system, but did not mention about the study period. | partly-did not mention about handling of loss to follow due to death. | yes | partly-though it mentioned about 30-day readmission, however, it did not contain description of definition of readmission outcome | yes | yes | Low |
| Goyal et al, 2019 | yes | yes | yes | yes | Parly, did not include pre-index utilization (such as length of stay) | yes | High |
| Garcia-Acre et al, 2018 | yes | yes | yes | yes | Yes | yes | High |
| Mortazavi et al, 2016 | Parly, described the main diagnosis of patients, but did not describe the population settings (single, multiple, state or national level?), and did not mention about study period. | yes | yes | yes | Yes | yes | High |
| **Study** | **Study Population*** | **Study Attrition†** | **Prognostic Measure‡** | **Outcome Measurement§** | **Study Confoundingǂ** | **Statistical Analysis and Reporting•** | **Quality**  **Assessment** |
| Frizzell et al, 2017 | yes | yes | yes | yes | Yes | yes | High |
| Eckert et al, 2018 | yes | yes | yes | yes | Yes | yes | High |
| Lodhi et al, 2017 | yes | partly-did not mention about handling of loss to follow due to death. | yes | no, did not provide timing of readmission outcome and did not provide the definition of readmission outcome | Parly, did not describe well about the predictor variables and did not include the number of candidate variables included for analysis | yes | Low |
| Jamei et al, 2017 | yes | yes | yes | yes | Yes | yes | High |
| Welchowski et al, 2016 | yes | yes | yes | yes | Yes | yes | High |
| Wang et al. 2018 | yes | yes | yes | yes | Yes | yes | High |
| Hopkins et al, 2019 | yes | party-did not mention % of patients have the readmission related information during 30 days follow up. | yes | yes | Yes | yes | High |
| Golas et al, 2018 | yes | yes | yes | yes | Yes | yes | High |
| Reddy et al, 2018 | yes | yes | yes | yes | Yes | yes | High |
| **Study** | **Study Population*** | **Study Attrition†** | **Prognostic Measure‡** | **Outcome Measurement§** | **Study Confoundingǂ** | **Statistical Analysis and Reporting•** | **Quality**  **Assesment** |
| Xiao et al, 2018 | Parly, described the main diagnosis of patients, but did not describe the population settings (single, multiple, state or national level?), and did not mention about study period. | yes | yes | yes | Yes | yes | High |
| Lin et al, 2019 | yes | yes | yes | yes | Yes | yes | High |
| Allam et al, 2019 | yes | yes | yes | yes | Yes | yes | High |
| Min et al. 2019 | yes | yes | yes | yes | Yes | yes | High |
| Rajkomar et al, 2018 | yes | yes | yes | yes | Yes | yes | High |
| * Study describes inclusion criteria for selecting patients, and for enrolled patients describes duration and severity of symptoms, and for enrolled patients describes demographics(at least age), and setings. † Data for hospital readmission outcome available for at least 80% of study populaton at the time of measurement. † Data for hospital readmission outcome available for at least 80% of study populaton at the time of measurement. ‡ Study describes appropriate methods for measuring prognostic factors. § Study describes reproducible and approproate methods to define and identify readmission; transfers and deaths during index hospitalization were excluded. ǂ Study includes at least the following predictors: demographics, length of stay, cormorbidities. • Study includes methods of validation. | | | | | | | |
